# Preparation of pH-sensitive nanogels bioconjugated with shark antibodies (VNAR) for targeted drug delivery with potential applications in colon cancer therapies

**DOI:** 10.1371/journal.pone.0294874

**Published:** 2024-01-19

**Authors:** Lizbeth A. Manzanares-Guevara, Jahaziel Gasperin-Bulbarela, Olivia Cabanillas-Bernal, Monserrat Renteria-Maciel, Angel Licea-Claverie, Eugenio R. Méndez, Alexei F. Licea-Navarro

**Affiliations:** 1 Biomedical Innovation Department, Centro de Investigación Científica y de Educación Superior de Ensenada (CICESE), Ensenada, Baja California, México; 2 Centro de Graduados e Investigación en Química, Tecnológico Nacional de México/Instituto Tecnológico de Tijuana, Tijuana, Baja California, México; 3 Applied Physics Division, Centro de Investigación Científica y de Educación Superior de Ensenada (CICESE), Ensenada, Baja California, México; University of Newcastle, AUSTRALIA

## Abstract

Cancer is the second leading cause of death worldwide. To combat this disease, novel and specialized therapeutic systems are urgently needed. This is the first study to explore a system that combines shark variable domain (Fv) of new antigen receptor (VNAR) antibodies (hereinafter VNARs), PEGylated nanogels (pH-sensitive poly(*N*,*N*-diethylaminoethyl methacrylate, PDEAEM), and the anticancer drug 5-fluorouracil (5-FU) to explore its potential applications in colon cancer therapies. Nanogels were functionalized in a scalable reaction with an *N*-hydroxysuccinimide (NHS)-terminated polyethylene glycol derivative and bioconjugated with shark antibodies. Dynamic light scattering measurements indicated the presence of monodispersed nanogels (74 to 236 nm). All systems maintained the pH-sensitive capacity to increase in size as pH decreased. This has direct implications for the release kinetics of 5-FU, which was released faster at pH 5 than at pH 7.4. After bioconjugation, the ELISA results indicated VNAR presence and carcinoembryonic antigen (CEA) recognition. *In vitro* evaluations of HCT-116 colon cancer cells indicated that functionalized empty nanogels are not cytotoxic and when loaded with 5-FU, the cytotoxic effect of the drug is preserved. A 15% reduction in cell viability was observed after two hours of contact with bioconjugated nanogels when compared to what was observed with non-bioconjugated nanogels. The prepared nanogel system shows potential as an effective and site-specific nanocarrier with promising applications in *in vivo* studies of colon cancer therapies.

## 1. Introduction

Developing effective cancer treatments and a possible cure for cancer constitute some of the most difficult challenges facing modern medicine due to the complex nature of the disease. These challenges mainly stem from the diversity of cancer cells, tumor heterogeneity, adverse drug-related effects, and the drug-resistance developed by malignant cells [1]. Each of these obstacles must be strategically addressed when developing effective treatments for particularly deadly cancers like colorectal cancer, which is the third most common cause of cancer deaths worldwide after breast and lung cancer [2,3]. Chemotherapy, which relies on chemical agents to kill cancer cells, is one of the most important tools available to treat cancer. However, in its usual form, chemotherapy is not sufficiently selective because it kills an unacceptable number of healthy cells while destroying malignant cells [4,5]. For example, the anticancer drug 5-fluorouracil (5-FU), which has proven to be effective in treating colorectal cancer, has limited clinical applications due to its toxicity to healthy cells and the resistance that cancer cells develop to this drug over time [6].

A possible solution to overcome both of these obstacles can be generated by enhancing the selectivity of chemotherapeutic agents by exploiting the differences between healthy and neoplastic cells. To this end, if cancer drugs can be specifically directed and released within tumor cells, the toxicity associated with chemotherapies would be markedly reduced. The resulting increase in selectivity would allow chemotherapy drugs to be safely administered in higher doses, thus improving their effectiveness [1]. Nonetheless, developing curative cancer chemotherapies constitutes an ultimate pharmacological goal that must also be addressed with future drug discoveries to change the face of this devastating disease [7].

Nanocarriers are nanometric bodies that can be used as transport modules for other substances such as cancer drugs. Nanohydrogel carriers developed from natural and synthetic polymers show great potential for drug delivery due to their loading capacity and biocompatibility. However, some obstacles must be overcome before nanohydrogels are approved for clinical use. An ideal drug carrier should be able to target specific sites to release its cargo while evading elimination by the immune system. Although promising formulations exist, most of these do not meet all biological criteria [8], which justifies the need for further studies to develop improved nanocarriers. Polymerizable PEGMA macromonomers stabilize droplets of water-insoluble DEAEM (at neutral pH) to yield a series of core-shell nanogels with different characteristics. Some important aspects should be considered for their potential application as drug nanocarriers. An ideal nanogel carrier for drug delivery should meet a few criteria, including small particle size (10–200 nm), biodegradability, biocompatibility, prolonged blood circulation time, high capacity for drug or enzyme loading or entrapment, and immune system protection [9].

Antibodies constitute an interesting component of cell signaling given that they function as antennas in the presence of their antigens. Most conventional antibodies are composed of two heavy chains linked to two light chains by two disulfide bonds, although other interesting antibody structures can be found in various organisms. For example, sharks produce some unusual antibodies that are only composed of heavy chains. The variable domain (Fv) of new antigen receptor (VNAR) antibodies from sharks can also be designated as a single domain. VNAR antibodies (hereinafter VNARs) are usually produced as recombinant proteins [4] VNARs from sharks exhibit special characteristics when compared to those of conventional antibodies, such as small size (12–15 kDa), thermal and chemical stability, and great tissue penetration, which make these antibodies promising alternative sources for therapeutic and diagnostic agents [10].

Studies that have combined nanogels and antibodies are uncommon. Moreover, studies combining marine-derived antibodies, such as VNARs, and nanogels have not been published to the best of our knowledge, although similar studies have employed monoclonal antibodies. For example, Adamo et al. produced (*N*-vinyl pyrrolidone)-based-nanogels conjugated with monoclonal antibodies for active targeting purposes and evaluated the uptake of immuno-functionalized nanogels in the endothelial cell line ECV304 through intracellular localization studies. These authors found that the use of immuno-conjugated nanogels resulted in faster uptake when compared with the uptake rates of unconjugated nanogels. Given that immuno-conjugated nanogels can recognize specific cell types in heterogeneous systems, they are promising systems for targeted drug delivery [11].

**Scheme 1:**
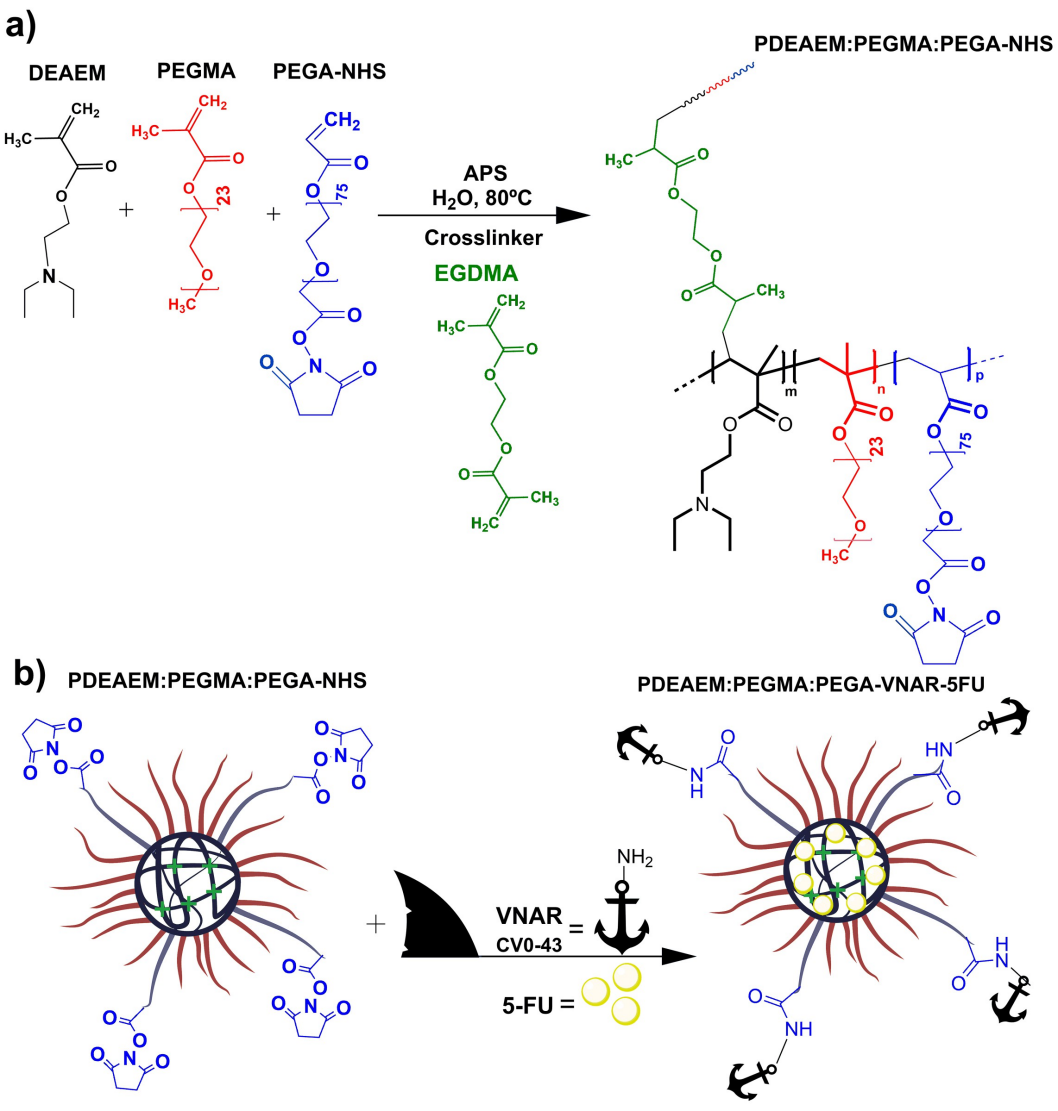
Nanogels synthesized by surfactant-free emulsion polymerization (SFEP): a) Chemical structures for PDEAEM:PEGMA:PEGA-NHS nanogels, APS is (NH_4_^+^)_2_(S_2_O_8_)^2-^, b) Nanogels bioconjugated with shark antibodies: PDEAEM:PEGMA:PEGA-VNAR-5FU. Poly(*N*,*N*-diethylaminoethyl methacrylate) (PDEAEM, black), poly(ethylene glycol) methacrylate (PEGMA, red), acrylate-PEG3500-NHS (PEGA-NHS, blue), variable domain of new antigen receptor (VNAR, anchor), ethyleneglycol dimethacrylate (EGDMA, green), and 5-fluorouracil (5-FU, anticancer drug, yellow circle).

Novel therapeutic systems and strategies to cure and treat cancer, such as those that employ nanogel-conjugated VNARs, are urgently needed. To this end, a simple synthetic strategy is presented in this study to prepare poly(*N*,*N-*diethylaminoethyl methacrylate) (PDEAEM)-based nanogels by one-pot surfactant-free emulsion polymerization (SFEP) using commercially available poly(ethylene glycol) methyl ether methacrylate (PEGMA) as a polymerizable stabilizer and *N*-hydroxysuccinimide (NHS), which was responsible for the functionalization of poly(ethylene glycol)-acrylate (PEGA) as the shark VNAR anchor. The nanogels were conjugated with the VNAR CV0-43, as this antibody can recognize the carcinoembryonic antigen (CEA) present in colon cancer cells [10] Nanogel preparation for bioconjugation and VNAR anchoring is depicted in Scheme 1.

## 2. Materials and methods

### 2.1. Materials

*N*,*N*-(Diethylamino)ethyl methacrylate (DEAEM, 99%) was purified by distillation under reduced pressure prior to use. PEGMA (MW = 950), Acrylate-PEG3500-NHS (PEGA-NHS), and ethylene glycol dimethacrylate (EGDMA, 98%) were purified by passing through an inhibitor remover column for hydroquinones. Ammonium persulfate (APS, 98%) and 5-fluorouracil (5FU, 99%) were used as received. Reagents were purchased from Sigma-Aldrich (St. Louis, MO, USA), and commercial grade distilled water (Sparkletts, Lakeside, CA, USA) was used. Antibody anti-HA-HRP High affinity (Clone 3F10, Roche, Nutley, NJ, USA) was purchased from Sigma-Aldrich. 1-Step™ Turbo Pierce™ TMB solution was purchased from Thermo Fisher Scientific (Waltham, MA, USA). Cell line HCT-116 (CCL-247) was obtained from ATCC (Manassas, VA, USA), and DMEM high glucose media and fetal bovine serum (FBS) were purchased from Biowest (Riverside, MO, USA). McCoy 5a media was obtained from Corning (New York, NY, USA), and cell culture antibiotic/antimycotic solution was obtained from Sigma-Aldrich. CellTiter 96® AQueous One Solution Cell Proliferation Assay (MTS) was purchased from Promega (Madison, WI, USA).

### 2.2 PDEAEM:PEGMA:PEGA-NHS nanogel synthesis

Functionalized PDEAEM:PEGMA:PEGA-NHS nanogels were obtained by one-pot Surfactant-Free Emulsion Polymerization (SFEP). In a typical experiment, a reaction mixture containing DEAEM, PEGMA, PEGA-NHS, and EGDMA (2 mol% with respect to DEAEM content) in 50 mL of distilled water was bubbled with nitrogen for 30 min (at room temperature) with magnetic stirring before the polymerization reaction. The reaction mixture was then poured inside a 0.5-L jacketed glass reactor (Atlas Potassium System, Syrris, Royston, UK) containing 240 mL of deionized and degassed water (nitrogen atmosphere) at 80°C and vigorously stirred (500 rpm). The initiator APS (0.192 g, 0.904 mmol) was dissolved in 10 mL of deionized water and immediately added to the reaction vessel to act as a thermal initiator. The resultant concentration in the reactor was always 5 g/L. The polymerization process was stopped after 1 h by cooling. The resulting dispersions were purified by dialysis (Spectra/Por® dialysis membrane, MWCO: 12–14 kDa; Spectrum Laboratories, Rancho Dominguez, CA, USA) against deionized water for 10 days (daily water changes). Finally, samples were frozen and lyophilized in a FreeZone 4.5-Liter Freeze Dry System (Labconco, Kansas City, MI, USA). The products were stored in a refrigerator until use. The synthesis of non-functionalized nanogels (without PEGA-NHS) was performed similarly and is described in detail in a previous study from our research group [12].

#### 2.2.1 Characterization

The chemical composition of the PDEAEM-based nanogels was quantified by proton nuclear magnetic resonance (^1^H-NMR; AVANCE III HD NMR 400 MHz equipment, Bruker, Billerica MA, USA) using deuterated chloroform (CDCl_3_). The chemical shifts are reported in ppm using tetramethylsilane (TMS) as the internal standard. Infrared (IR)-spectra were obtained using a 4700 Fourier transform infrared (FTIR) spectrometer (JASCO Corporation, Tokyo, Japan). The size distribution of the nanogels was obtained by dynamic light scattering (DLS) using a Zetasizer Nano ZS (ZEN3690; Malvern Instruments, Worcestershire, UK) equipped with a red laser (630 nm). The measurement angle was 90°, and dialyzed and redispersed samples were analyzed. The hydrodynamic diameter (Dh) and polydispersity index (PDI) were calculated using Malvern Instruments dispersion technology software based on CONTIN analysis and the Stokes–Einstein equation for spheres. For D_h_, the average value of three measurements is reported. To evaluate pH sensitivity, measurements were conducted at 20°C from pH 5 to pH 8 using a buffer solution at each pH value. The zeta potential (ζ) was also measured using the same Zetasizer Nano ZS equipment by laser doppler microelectrophoresis. Measurements were performed on folded capillary cells at 20°C. UV-Vis absorption spectra of selected nanogels were recorded using a UV–Vis Cary 100 spectrophotometer (Varian, Palo Alto, CA, USA) at room temperature. Differential scanning calorimetry (DSC) equipment (Q2000, TA Instruments, New Castle, DE, USA) was used in a modulated DSC mode. Each sample was cooled to -10°C and isothermally maintained for 5 min. Then, the temperature was modulated to ± 0.5°C every 60 s and heated 5°C min^-1^ until 200°C. The reported results correspond to the second cycle. Thermogravimetric analysis (TGA) was performed with a Discovery TGA analyzer (TA Instruments) using a heating ramp of 10°C min^-1^ from 35°C to 700°C in nitrogen atmosphere.

### 2.3 Loading of 5-FU into nanogels

To encapsulate 5-FU in PDEAEM:PEGMA and functionalized PDEAEM:PEGMA:PEGA-NHS nanogels, a nanogel dispersion was prepared in 10 mL of water with a 1:1 weight ratio of dry nanogel:5-FU, and the mixtures were stirred for 48 h at room temperature. Afterwards, the drug-loaded nanogels were dialyzed against distilled water to remove surface adsorbed and non-encapsulated drugs with a Spectra/Por® membrane (MWCO: 12–14 kDa). The loaded nanogels were frozen for 12 h and then freeze dried at -50°C and 0.05 mbar for 24 h. The drug-loaded nanogels were dispersed in PBS buffer at pH 7.4 and analyzed using a UV-Vis Cary 100 Spectrophotometer (Varian; 266 nm). 5-FU was loaded in the bioconjugated nanogels (PDEAEM:PEGMA:PEGA-VNAR) at 4°C, which were then dialyzed against PBS 7.4 in Slide-A-Lyzer Dialysis Cassettes (7K MWCO). The drug loading (%DL) and encapsulation efficiency (%EE) were calculated using Eqs (1) and (2), respectively [13]:

$$DL(\%)=(\frac{MDng}{Mng+MDng})x100$$ (1)

and

$$\mathrm{EE}(\%)=\left(\frac{MDng}{MD}\right)x100,$$ (2)

where MDng is the drug mass in the nanogels, Mng is the nanogel mass, and MD is the drug mass in the feed.

### 2.4. *In vitro* 5-FU release profile

*In vitro* release studies were performed under sink conditions at controlled temperature and agitation using a Precision SWB 15 shaking bath (Thermo Scientific). The operating conditions were established as follows. First, 37°C and pH 7.4 were selected as conditions that mimic the environment of a healthy body, and 37°C and pH 6 were selected as conditions that mimic the tumor microenvironment. In both cases, constant stirring was maintained at 90 rpm. Typically, 5 mg of drug-loaded nanogels were dispersed in 3 mL of the corresponding buffer and then placed in a Spectra/Por® dialysis bag (MWCO: 1 kDa), which was subsequently immersed in 300 mL of the release medium at the desired temperature. At predetermined time intervals, an aliquot (3 mL) was withdrawn, and 3 mL of clean PBS was added to keep the volume of the release medium constant. The amount (%) of free 5-FU was estimated using a calibration curve (S1 Fig in S1 File). The percentage of drug released (%DR) was calculated with Eq (3), [13]:

$$DR(\%)=\left(\frac{mass\;of\;drug\;released\;from\;nanogels}{mass\;of\;total\;drug\;loaded\;in\;nanogels}\right)x100.$$ (3)

Drug release was evaluated with UV-Vis spectroscopy (266 nm).

### 2.5 VNAR expression and purification

#### 2.5.1 Expression

The VNAR CV0-43 was obtained from the periplasm of *E*. *coli* BL21 (DE3) cells. A single colony of interest was placed in 50 mL of TB medium containing carbenicillin and incubated overnight at 37°C with agitation (250 rpm). The culture was used to inoculate 950 mL TB medium containing carbenicillin and incubated at 37°C with agitation (250 rpm) until the culture reached an OD600 value of 0.8. Induction was performed with 0.5 mM IPTG (Sigma Aldrich) and incubation for an additional 8 h. The culture was centrifuged at 6 000 x *g* for 30 min. The cell pellet was used for the periplasmic extraction of the protein according to the QIAexpressionist manual (QIAGEN) with 40 mM of imidazole. After extraction, the supernatant was centrifuged at 14 000 x *g* for 30 min at 4°C, and 10% glycerol was added before purification.

#### 2.5.2 Purification

Immobilized metal affinity chromatography (IMAC) was used for protein extraction in an ÄKTA FPLC-UPC 900 system and HisTrap FF 5 mL column (GE Healthcare, Chicago, IL, USA). The supernatant was filtered with a cutoff membrane (0.2 μm), and the column was equilibrated with NPI40 binding buffer (NaCl 500 mM/NaH_2_PO_4_ 4.92 mM/Na_2_HPO_4_ 15.08 mM/Imidazole 50 mM, pH 7.4). Then, the periplasmic extract was loaded in the column at a constant flux of 1 mL min^-1^. After sample loading was complete, 15 mL of NPI40 binding buffer was added, followed by 25 mL of NPI50 wash buffer (NaCl 500 mM/NaH_2_PO_4_ 4.92 mM/Na_2_HPO_4_ 15.08 mM/Imidazole 50 mM, pH 7.4). The protein was eluted with 30 mL of NPI500 elution buffer (NaCl 500 mM/NaH_2_PO_4_ 4.92 mM/Na_2_HPO_4_ 15.08 mM/Imidazole 500 mM, pH 7.4), which was collected in 5-mL fractions.

#### 2.5.3 Purification analysis

Fractions containing purified protein were analyzed by tricine sodium dodecyl sulphate-polyacrylamide gel electrophoresis (SDS-PAGE), and a sample of each fraction of interest was denaturalized using loading buffer [Tris-HCl 125 mM pH 6.8/SDS 4% (w/v)/glycerol 20% (v/v)/β-mercaptoethanol 10% (v/v)/bromophenol blue 0.004% (w/v)], boiled for 10 min at 95°C, and loaded into two gels (12% polyacrylamide with SDS and tricine). The tricine SDS-PAGE was run at 50 mA for 2 h. One gel was stained with coomassie blue solution, and the other gel, which was used for western blot analysis, was transferred to a nitrocellulose membrane (BIORAD, Hercules, CA, USA) for 1 h at 20 volts using a trans clot semi-dry electrophoretic transfer cell (BIORAD). The membrane was blocked with 8% milk-PBST overnight at 4°C. The blocking solution was discarded, and high affinity anti-HA-HRP (ROCHE, Basel, Switzerland) diluted 1:3500 in 3% milk-PBST was added. The reaction was incubated for 1 h at room temperature with agitation. The antibody solution was discarded, and the membrane was washed in agitation for 10 min with PBST three times. The membrane was incubated in a Pierce™ DAB solution (Thermo Scientific) until color developed.

#### 2.5.4 CV0-43 purification

A Spin-X® UF protein concentrator (Corning, 5K MWCO) was used to concentrate and desalt the purified protein. The protein remaining on the tube was washed with PBS (pH 7.4) and then quantified by UV-Vis using a nanodrop 2000 spectrophotometer (Thermo Scientific). The concentration was 350 μg/mL. The protein was filtered with a cutoff membrane (0.2 μm) and stored at -20°C until use.

### 2.6 Bioconjugation of nanogels

#### 2.6.1 Bioconjugation of nanogels with GFP and Folic acid

The nanogels were bioconjugated using NHS chemistry that generates coupling of the amino groups (GFP and FA in this case). A general synthetic procedure was followed as described below. First, 10 mL of the reaction mixture was prepared using 9.5 mL of functionalized nanogels (2 mg/mL in PBS at pH 8) and 0.5 mL of GFP or FA (1 mg/mL). The reaction was conducted at room temperature for 12 h in the dark with constant stirring. The product was purified by dialysis (Spectra/Por® dialysis membrane, MWCO: 50 kDa) for 21 h against 300 mL of PBS (pH 7.4). Then, it was analyzed by UV-Vis and fluorescence. Other biological tests were also performed and are described in sections 2.7–2.8.

#### 2.6.2 Bioconjugation of nanogels with shark VNARs

The nanogels were bioconjugated using NHS chemistry that generates coupling of the amino groups found in proteins (shark antibodies in this case). A general synthetic procedure for bioconjugated nanogels (PDEAEM:PEGMA:PEGA-VNAR) was followed as described below. First, 3 mL of the reaction mixture was prepared in a sterile vial using 1.5 mL of functionalized nanogels (N3) (2 mg/mL in PBS at pH 8 passed through a 0.22-μm filter) and 1.5 mL of VNAR (CV0-43, 350 μg/mL). The reaction was conducted at 4°C for 8 h in the dark with constant stirring. The product was purified by dialysis (Spectra/Por® dialysis membrane, MWCO: 50 kDa) for 21 h against 300 mL of PBS (pH 7.4). Then, it was analyzed by UV-Vis using a nanodrop 2000 spectrophotometer (Thermo Scientific). Other biological tests were also performed and are described in sections 2.7–2.8.

### 2.7 ELISA assays

To evaluate VNAR expression, 50 μl/well of sample were loaded in duplicate. The samples were incubated for 2 h at 37°C. Blocking was performed with 200 μl/well of 8% milk-PBS, followed by incubation for 1 h at 37°C. Four washes with PBS (200 μl/well) were performed to remove sediment. Then, 50 μl/well of anti-HA-HRP antibody 1:2500 in 3% milk-PBS was added, followed by incubation overnight at 4°C. Five washes were then performed with 0.5% PBST (0.5% Tween 20, 200 μl/well) to remove antibodies unbound to the HA tag. Then, 50 μl of TMB turbo ELISA was added at room temperature, followed by incubation for 30 min at 37°C. To stop the reaction, 50 μl of 10% HCl was added to each well.

For the CEA recognition assay, 50 μl/well of 5 ng/μl CEA were loaded in triplicate and incubated for 2 h at 37°C. Blocking was performed with 200 μl/well of 8% milk-PBS, followed by incubation overnight at 4°C. Five washes with PBS (200 μl/well) were performed to remove sediment. Then, 50 μl/well of the final product (nanogel N3 bioconjugated to VNAR [CV0-43]) was added at a concentration of 175 μg/mL (diluted 1:1 in PBS) as a control. Non-bioconjugated nanogel N3 (1 mg/mL) and milk (8%) was used as a negative control and incubated for 2 h at 37°C. Then, 50 μl/well of anti-HA-HRP antibody 1:3000 in 3% milk-PBS was added and incubated for 1 h at 37°C. Five washes with 0.5% PBST (0.5% Tween 20, 200 μl/well) were performed to remove antibodies unbound to the HA tag. A total of 50 μl of TMB turbo ELISA was added at room temperature, followed by incubation for 30 min at 37°C, and 50 μl HCl (10%) was added to the TMB solution of each well. ELISAs were measured spectroscopically at 450 nm with an EPOCH 96-well microplate reader (BioTek, Winooski, VT, USA).

### 2.8 Cell viability assay

Human colon cancer cells (HCT-116) were cultured in McCoy 5a medium supplemented with 10% FBS and 1% antibiotic/antimycotic solution in a humidified atmosphere with 5% CO_2_ at 37°C. Cells were seeded into a 96-well plate at a density of 5,000 cell/well and incubated for 24 h. Then, the media was replaced for the different treatments (diluted in fresh media) and incubated for another 2 or 12 h. Subsequently, the cells were washed and replaced with fresh media and incubated up to 24 h. The treatments that were evaluated were empty nanogels (N3), 5-FU (20 μg/mL), nanogels loaded with 5-FU (N3-5-FU), and nanogels loaded with 5-FU and functionalized with VNAR CV0-43 (N3-VNAR-5FU). The concentration of 5-FU in the nanogel solution was standardized to 20 μg/mL. Cell viability was evaluated with MTS. For this, the cells were washed with PBS, and 10% MTS solution diluted in fresh media was added. Then, cells were incubated for 2–3 h. Finally, absorbance was measured at 492 nm.

## 3. Results and discussion

### 3.1 Preparation of PDEAEM:PEGMA:PEGA-NHS nanogels

In this study, two types of nanogels were used: PDEAEM:PEGMA nanogels without NHS (synthesis described in our previous study [10], samples N0 and N01 in Table 1) and PDEAEM:PEGMA nanogels with PEGA-NHS (described in section 2.1) to anchor antibodies or molecules with available primary amino groups (samples N1 to N6 in Table 1).

**Table 1:** General characteristics of PDEAEM-based nanogels.

Key	DEAEM:PEGMA:PEGA initial content (Weight ratio)	PDEAEM:PEG (Weight ratio) by ^1^H-NMR	D_h_ (nm)	PDI	ζ potential (mV) at pH 7	DL/EE % (5FU)
**Non-NHS functionalized** (PEGMA950)^a^						
N0	60:40:0	20:80	74	0.167	+10.1	15/26.2
N01	70:30:0	25:75	90	0.097	+13.4	-
**NHS Functionalized** (PEGMA950 +PEGA3500-NHS)^a^						
^b^N1	70:0:30	-----	236	0.100	+3.70	-
N2	70:28:2	32:68	102	0.031	+5.48	21/28.6
N3	70:27:3	28:72	106	0.059	+5.18	17/27.0
N4	70:26:4	35:65	103	0.057	+4.30	19/27.8
N6	70:24:6	30:70	84	0.022	+3.05	14/25.8

^a^EGDMA 2%, and APS 3% (mol% with respect to DEAEM).

^b^Without PEGMA950, scaled-down (synthesis in 10 mL of distilled water).

The nanogels were characterized by DLS, ^1^H-NMR, IR, DSC, and TGA (S2–S9 Figs in S1 Fig S1 File). Based on the ^1^H-NMR analysis, the incorporation of PDEAEM and PEGMA+PEGA in the final products was corroborated, and the composition ratios are shown in Table 1. However, the characteristic NHS signal corresponding to hydrogens adjacent to carbonyls in the succinimide ring overlapped with the PDEAM signal at ~ 3.0 ppm (S5 Fig in S1 Fig S1 File) so that PEGA content was not determined separately by ^1^H-NMR. A similar case was observed in the infrared spectrum where the C-O (1100 cm^-1^) and C = O (ester, 1721 cm^-1^) stretch bands overlapped in the PEGMA950 and PEGA3500 compounds. In addition, a small signal can be observed at 1815 cm^-1^ for N2 corresponding to the C = O of the ester in NHS [14], which was not observed in the non-functionalized N01 nanogel. Through DSC, one thermal transition was observed for each nanogel: a sharp crystalline melting transition peak corresponding to PEG units. Some differences were observed in the PEG melting temperature, with temperatures of 32°C, 39°C, and 52°C for nanogels N01 (only with PEGMA950), N2 (PEGMA950, major proportion,+PEGA 3500), and N1 (only PEGA3500 and not PEGMA950), respectively. These differences in melting temperature can be attributed to the incorporation of PEGA3500 into the structure of the PDEAEM:PEGMA:PEGA3500-NHS nanogels (S6 Fig in S1 File). A slight trend of increasing melting temperature was observed among the functionalized nanogels (N2, N3, and N4) as the PEGA3500-NHS initial content increased during nanogel synthesis (S7 Fig in S1 File). The presence of the crystalline peak is proof of the presence of tethered PEG chains in the PDEAEM-based nanogels, which supports a possible core-shell morphology. Based on the TGA-analysis, the main decomposition temperature of the PDEAEM-based nanogels was ~ 400°C (S9 Fig in S1 File). As such, the nanogels are thermally stable and offer a synthetic possibility of changing the cross-linking agent to improve biodegradability, which has been explored in previous studies [9,12–15].

**Fig 1:**
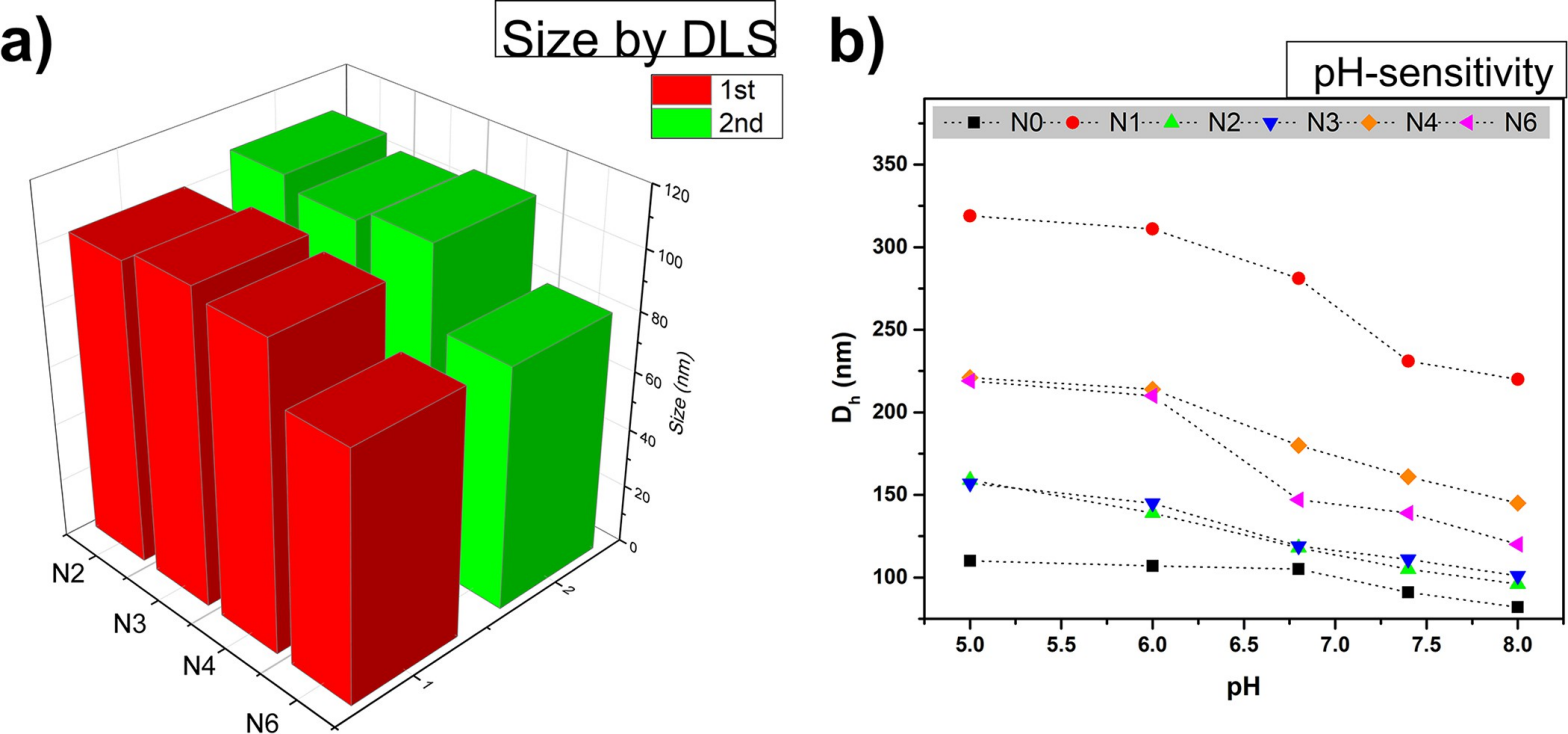
Sizes of the PDEAEM:PEGMA:PEGA-NHS nanogels determined by dynamic light scattering (DLS). a) Synthesis reproducibility and b) pH-responsive behavior of the nanogels.

Nanogel properties can be further tailored by altering the crosslinking density, by introducing specific functional groups, and by using stimuli-responsive constituents. To this end, PDEAEM:PEGMA:PEGA-NHS nanogels of controlled sizes (74 to 236 nm) were synthesized via PEGMA-mediated SFEP to increase their potential applications (Table 1). All nanogels show a positive surface charge (ζ-potential) at pH values less than 8 due to the effect of PDEAEM which is cationic as reported previously [15], non-NHS functionalized nanogels have a higher positive charge respect to NHS functionalized, the decrease in surface charge refers to the effect of NHS (S1 Table). Furthermore, monodisperse particles were obtained with the ability to redisperse in aqueous medium after lyophilization in concentrations up to 3 mg/mL and the colloidal dispersions of nanogels remained stable for more than a year at room temperature without observing macroscopic precipitation or aggregation with the naked eye. Two possible mechanisms for the colloidal stability of these nanogels are the electrostatic stabilization associated with the cationic segment and the steric stabilization provided by the PEG units. In addition, reactions N2 to N6 were reproducible and did not result in noticeable changes in nanogel size between two repetitions (Fig 1A). It is well known that one of the current challenges for the application of new drug delivery systems is precisely the aspect of large-scale production and maintenance of the size and composition of nanocarriers. For this reason, only a limited number of nanocarriers have currently been introduced into clinical trials. These reactions were also scalable (1X to 3X), and up to 0.5 g of product was obtained per reaction (S10 Fig in S1 File). Based on the gravimetric results, conversions were about 30 wt%. All nanogels exhibited responsive behavior and increased its size as pH decreased (Fig 1B) due to the protonation of the tertiary amino groups present in PDEAEM. This directly affects the release kinetics of 5-FU, as discussed later in section 3.2.

### 3.2 Evaluation of nanogel bioconjugation and 5-FU loading and release

**Fig 2:**
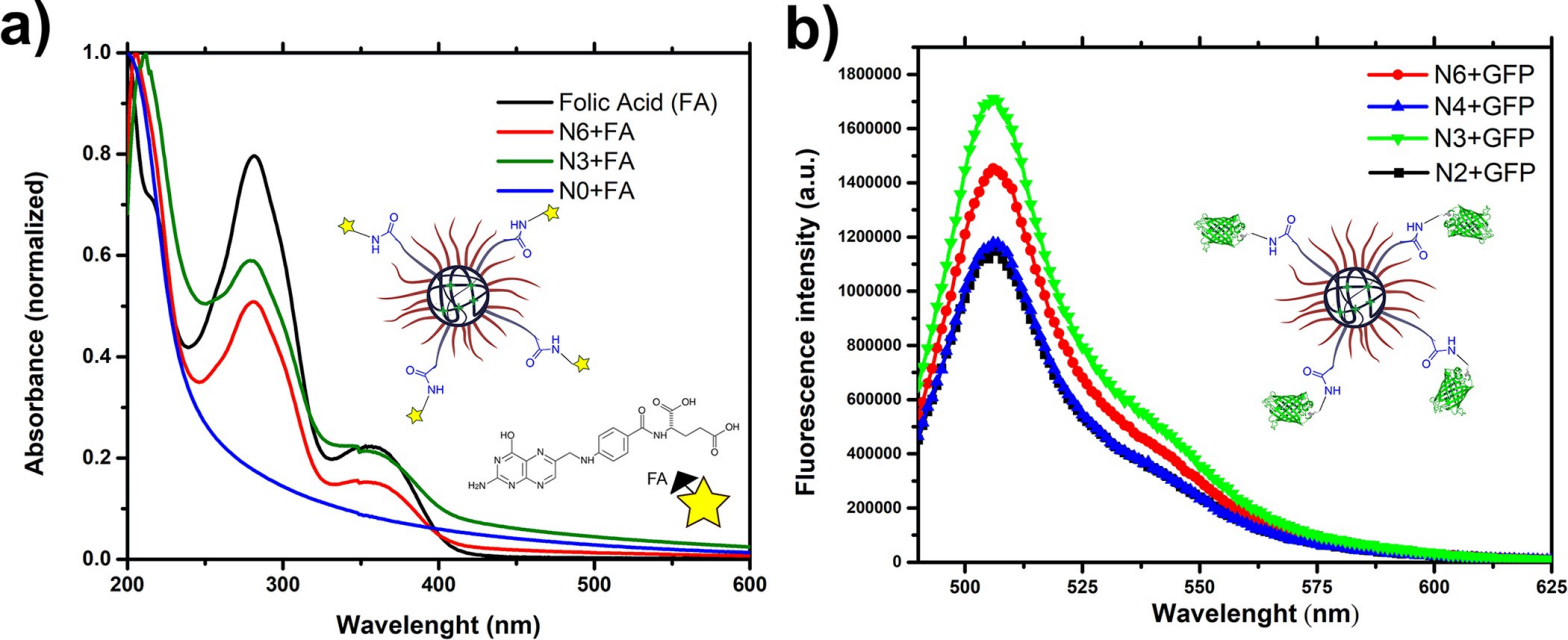
Model structures for bioconjugation of PDEAEM:PEGMA:PEGA-NHS nanogels. A) UV-Vis of folic acid-bioconjugated nanogels. B) Fluorescence emission spectra of GFP-bioconjugated nanogels.

Nanogels were functionalized with commercial PEGA-NHS, and the functionalization success was evident by the bioconjugation of the nanogels with shark VNARs. NHS ester chemistry is a well-known and favorable functionalization strategy to introduce new functionalities into polymeric nanoparticles and shows great potential for the conjugation of biomacromolecules to colloids due to the free amine groups present in different amino acids such as lysin [16]. The folic acid anchor containing a primary amino group was evaluated prior to bioconjugation with the antibody (Fig 2A). Folic acid is known to impart specific and high-binding affinity (*K*_*d*_, ~ 10^−10^ M) to folate receptors, which are overexpressed ~ 100–300 times in most cancer cells, including colon cancer cells [17] As such, these nanogels have potential use in further anticancer studies. Folic acid exhibits two absorption peaks at 295 and 358 nm [18]. The two absorption peaks of the functionalized products N3 and N6 were observed at 298 nm and 360 nm (N3) and 299 nm and 361 nm (N6). These peaks were not found in N0 (non-functionalized nanogels with PEGA-NHS; Fig 2A).

In the second model with green fluorescent protein (GFP)-bioconjugated nanogels, bioconjugation was evaluated by molecular fluorescence, and maximum fluorescence was detected at 505 nm for N2, N3, N4, and N5 (Fig 2b). Additional spectra for GFP and GFP-bioconjugated nanogels at PBS 7.4 can be seen in supporting information S11-S13 Figs in S1 File. This fluorescence was preserved while the sample was kept at 4°C and pH 8, which agrees with what has been reported in the literature [19]. GFP and related fluorescent proteins constitute a protein family with emission spectra that range from 442 to more than 650 nm. Fluorescent proteins range in size from 25 to 30 kDa and form internal chromophores that do not require cofactors or substrates to fluoresce. Fluorescent proteins generally have very high extinction coefficients (< 95,000) and very high quantum yields (< 0.8), which make these proteins very bright [19]. Taken together, these findings allowed us to anticipate that successful bioconjugation with shark antibodies would occur.

Controlled and targeted drug delivery systems can act for prolonged periods at tumor sites due to specific cell-surface interactions without affecting normal tissues [20]. Most tumors are characterized by an overexpression of cancer-specific antigen(s) or receptor(s) on their cell surfaces, which are essential for tumor cell growth. Therefore, the use of nanocarrier-based delivery systems to target these antigen(s) or receptor(s) is being extensively researched as an important treatment modality. To overcome current limitations, novel formulations of treatment modalities must be explored [21]. The nanogels in this study were bioconjugated with the methodology described in section 2.6.

**Fig 3:**
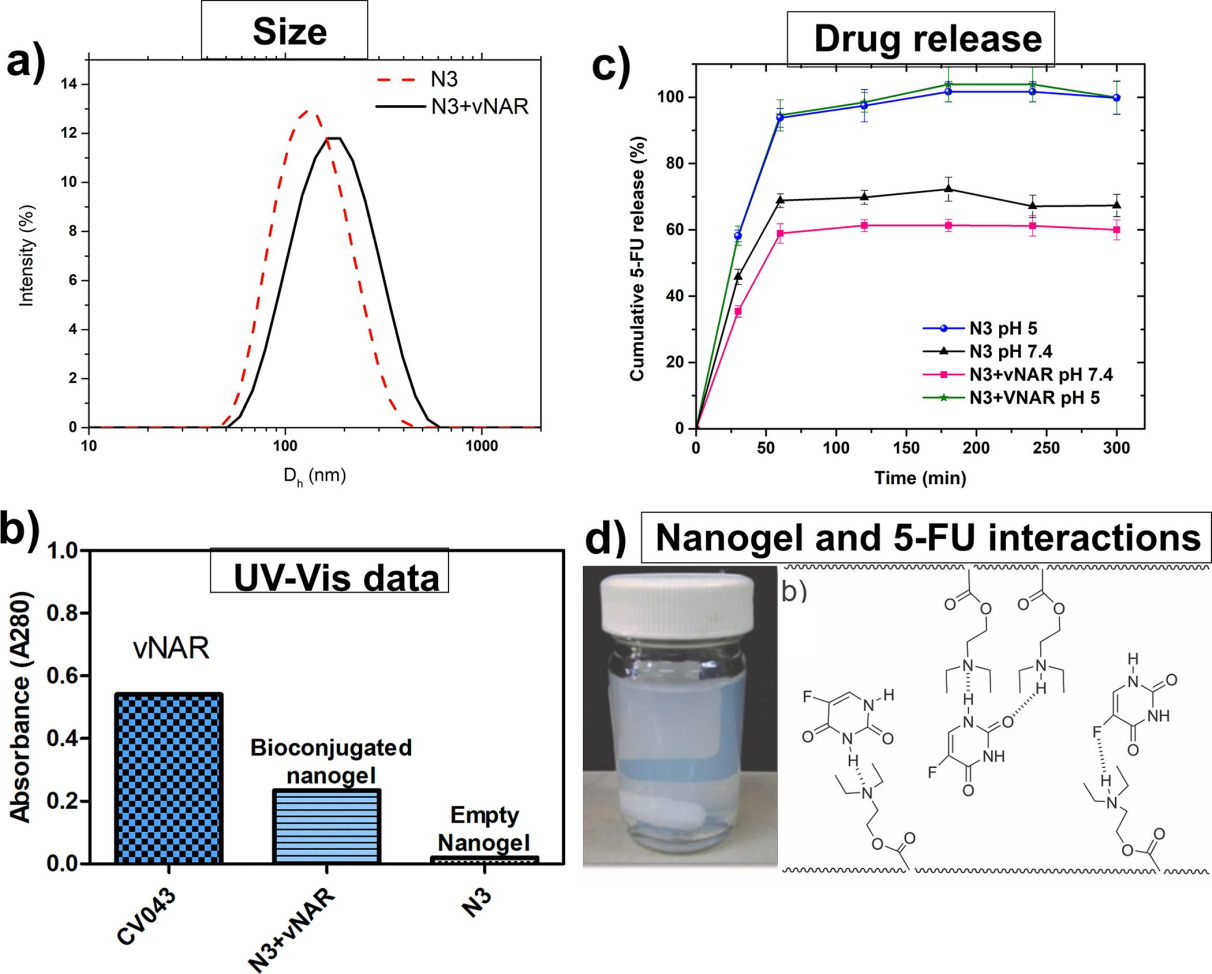
Analysis of functionalized nanogels (N3), VNAR-bioconjugated nanogels, and nanogels loaded with 5-FU. A) Size distribution by dynamic light scattering (DLS) measurements at pH 8, b) UV-Vis absorbance at 280 nm, c) cumulative 5-FU release at pH 7.4 and pH 5, and d) nanogel dispersion (N3+5FU, pH 7.4) and model of chemical interactions between PDEAEM-based nanogels and 5-FU.

Nanogel N3 was selected for further studies, and an increase in the size of the nanogel from 135 nm to 171 nm was observed by DLS when it was anchored to the VNAR (Fig 3A). Subsequently, several evaluations were conducted to confirm that the VNARs had been incorporated into the nanogels. For this, we employed UV-Vis analysis because it is well known that tryptophan (Trp) and tyrosine (Tyr) exhibit proteins (in our case antibodies) that absorb UV radiation in the range of 250–300 nm with maximum absorption at 280 nm, whereas histidine (His) shows weak absorption at 280 nm [22]. The bioconjugated nanogels (N3+VNAR) showed absorption at 280 nm. This contrasted with what was observed with the nanogels that were not conjugated with VNAR, which did not notably absorb in that region of the spectrum at the same concentration. This provides evidence of the incorporation of shark VNARs into the nanogels (Fig 3B).

Cocktails of cytotoxic chemotherapeutics are administered to treat colorectal cancer (i.e., 5-FU [23,24], trifluridine [24], tipiracil [24], irinotecan [25], and oxaliplatin [26]) both alone or in combination with targeted therapies. These chemotherapeutics with the exception of irinotecan, are hydrophilic (octanol/water partition coefficient [logP] values of less than 0.07) and are thus rapidly cleared from the bloodstream. Therefore, nanogels, which extend drug circulation time and improve tumor targeting, cell uptake, and the cytosolic delivery of hydrophilic chemotherapeutics, show promise for use in colorectal cancer therapies [26]. In this study, the nanogels were loaded with 5-FU in amounts ranging from 14 to 21%, and the efficiency of the loading process varied from 25 to 28%. Thus, the values were acceptable to continue with *in vitro* studies.

Normal blood pH for sustaining human life is ~ 7.4, whereas tumor tissues exhibit lower extracellular pH values of 6.5–7.2, which drop to 5.0–5.5 upon reaching the endosomes and lysosomes [27]. Therefore, *in vitro* drug release experiments were conducted under two pH conditions. Fig 3C shows the release of 5-FU under sink conditions, at 37°C, and at pH 5 and 7.4. In all cases, independent of functionalization or bioconjugation, accelerated release was observed in the first 60 min. This is beneficial if the nanocarrier is directed to an action site and rapid accumulation occurs at that site. After reaching the maximum release rate, release remained constant and was sustained up to 72 h (not shown). In addition, changes in the release rate were observed due to changes in pH. The release rate increased by 25% at pH 5 when compared to that at pH 7.4 with the non-bioconjugated nanogel N3. A similar change was observed in the bioconjugated nanogel N3+VNAR. In this case, release increased by 35% at pH 5 (Fig 3C) compared to that at pH 7.4. This pattern suggests that the nanogels expand at acidic pH and that 5-FU-nanogel interactions are weakened, which allows for drug diffusion. 5-FU does not possess ionizable functional groups, which means interactions with nanogels are limited to hydrophobic interactions with diethyl groups of PDEAEM at high pH values and to hydrogen bonding with partially protonated tertiary amines from PDEAEM at low pH values, as suggested by Mohamed et al. for 5-FU and other nanogel types [28]. At neutral pH, a combination of interactions is possible given that the acidity constant (pK_a_) of PDEAEM is close to 7 (Fig 3D).

With the aim of reducing antibody formats to minimal fragments that retain their antigen binding capacity, two unique antibody isotypes have been discovered that naturally contain a single domain for antigen binding. The first of these antibody isoforms is found in the blood serum of Camelidae. These are known as heavy chain antibodies (HCAbs). A second antibody isoform has been reported in cartilaginous fish such as sharks. This immunoglobulin with a new antigen receptor (IgNAR) is a homodimer composed of two heavy chains joined by disulfide bonds and lacks light chains. Each heavy chain contains five constant domains and a VNAR, which is responsible for antigen recognition. VNAR CV0-43, which was used in this study, was previously isolated in our laboratory from a synthetic library using a VNAR from the horn shark (*Heterodontus francisci*) as a scaffold. In a previous study, CV0-43 showed the highest levels of expression with an absorbance value at least twice that of bovine serum albumin (BSA), which was used as a negative control [10] In that study, the ability of CV0-43 to recognize CEA was also confirmed by ELISA. The expression and purification of VNAR is described in the supplementary material (S14 Fig in S1 File).

**Fig 4:**
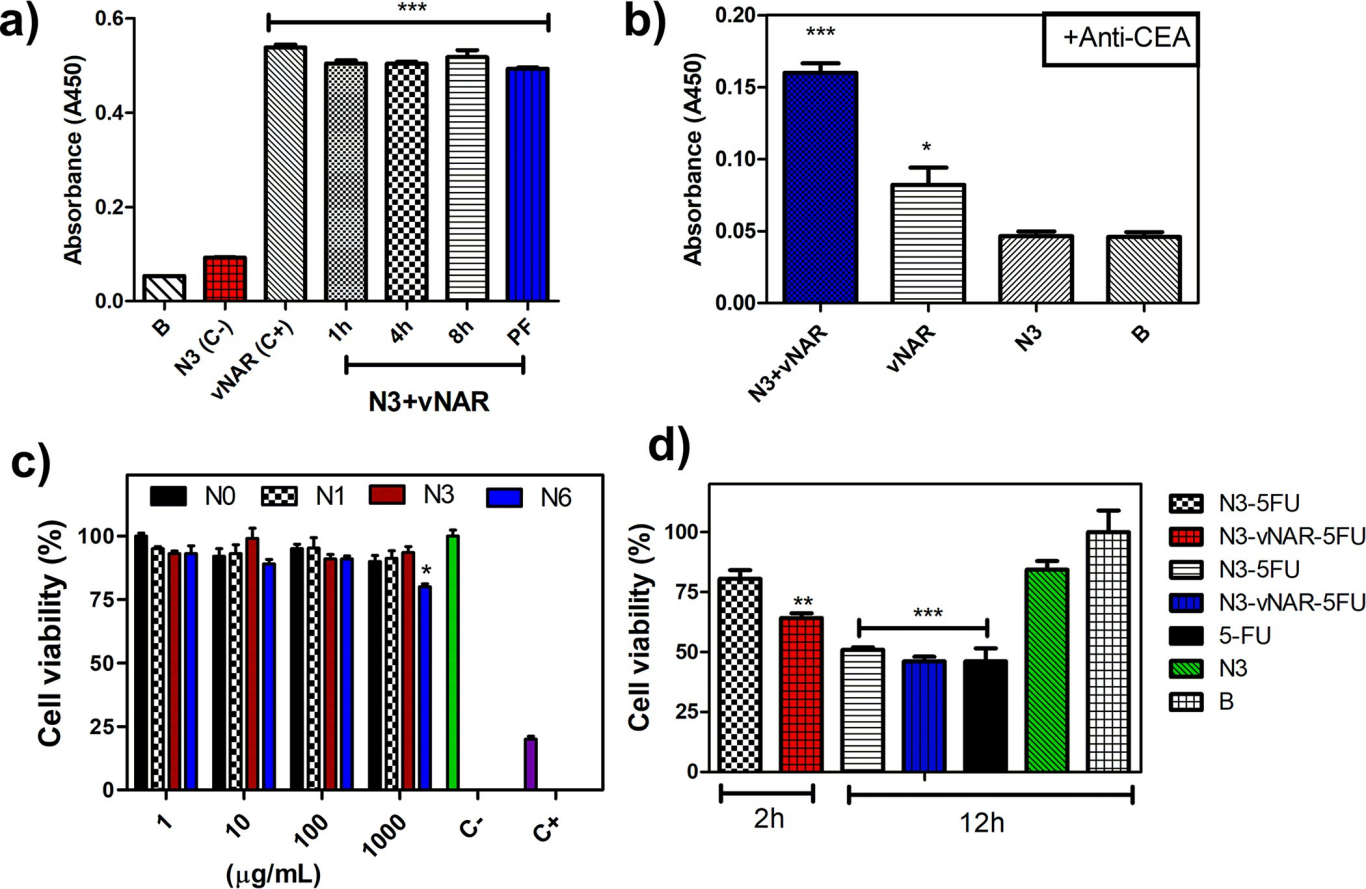
*In vitro* analysis of VNAR-bioconjugated nanogels (N3). A) ELISA of VNAR expression. On the horizontal axis: B (blank, Svelty milk 8%-PBS), N3 C- (nanogel without VNAR), C+ (CV043, 175 μg/mL), 1h (sample taken at hour 1 of the coupling reaction), 4h (sample taken at hour 4 of the coupling reaction), 8h (final sample taken at hour 8 of the coupling reaction), and PF (final product after dialysis [N3+VNAR]). B) ELISA assay of CEA recognition. On the horizontal axis: VNAR (CV043, 175 μg/mL), B (blank, Svelty milk 8%-PBS), c) cell viability by aqueous one solution cell proliferation assay (MTS) of empty selected nanogels at 24h, PBS (C-), DMSO 5% (C+), d) cell viability by MTS of 5-FU and nanogels containing 5-FU. On the horizontal axis: B (PBS 7.4), N3 (empty nanogels, 5-FU [20 μg/mL]), N3-VNAR-5FU (20 μg 5FU/mL), and N3-5-FU (20 μg 5FU/mL). ANOVA test, *p < 0.01, **p < 0.001, and ***p < 0.0001 versus N3 and PBS (C-). PDEAEM: poly(N,N-diethylaminoethyl methacrylate), PEGMA: poly(ethylene glycol) methyl ether methacrylate, PEGA-NHS: Acrylate-PEG3500-NHS, and CEA: carcinoembryonic antigen.

We corroborated VNAR expression in the purified product (N3+VNAR) after the bioconjugation reaction with ELISA (Fig 4A). The same samples were also evaluated by UV-Vis (280 nm). We found that 17% of what was initially available for the bioconjugation reaction was retained in the final product, equivalent to 30 μg of VNAR per 1 mg of nanogel (S15 Fig in S1 File) Another ELISA experiment was conducted to evaluate if the VNAR conjugated to the nanogel retained its ability to recognize CEA. Given that this ability was retained (Fig 4B), N3+VNAR is a suitable candidate for *in vitro* studies.

On the other hand, changes in the zeta potential were also observed, and the values measured by DLS were +3.87 mV (N3, nanogel only), -8.16 mV (VNAR, shark antibody), and -4.15 mV (N3+VNAR, bioconjugated nanogel). A change in the global charge of the particle can indicate antibody-nanogel bioconjugation. Previous studies have reported that the zeta potential may be used to measure the electrostatic potential at the double electrical layer surrounding a nanoparticle in solution. Nanoparticles with zeta potentials between -10 and +10 mV are considered relatively neutral, whereas nanoparticles with zeta potentials greater than +30 mV or less than -30 mV are considered strongly cationic or anionic, respectively.

Given that most cellular membranes are negatively charged, the zeta potential can influence the ability of a nanogel to permeate a membrane, with cationic particles generally displaying more toxicity associated with cell membrane disruption [29]. Based on the surface charge values, PDEAEM:PEGMA:PEGA-VNAR nanogels (N3) are considered neutral, which suggests that the observed cytotoxic effect may primarily be due to the interaction between VNAR and CEA that is expressed in HCT-116 colon cancer cells (based on the ATCC® database, 1 ng per 10^6^ cells/10 days) and not electrostatic interactions. *In vitro* studies on HCT-116 cells have shown that functionalized empty nanogels are not cytotoxic and when these are loaded with 5-FU, its cytotoxic effect is preserved (See IC_50_ of 5-FU in S16 Fig in S1 File). Cell viability in the presence of the empty nanogels was examined since it is reported that PDEAEM-containing nanogels show cytotoxicity depending on their concentration [30], Fig 4E shows the cell viability by proliferation assay (MTS) of empty nanogels at 24 h, and it can be seen that the systems are not cytotoxic up to a concentration of 1 mg/mL, so the effect on the decrease in cell viability is associated with the release of 5FU inside the cells as result of internalization of the nanogel due to the positive charge effect and the effect of VNAR affinity to CEA receptors.

Bioconjugated nanogels were found to decrease cell viability from 80% (without VNAR) to 65% (with VNAR) at 2 h. After 12 h of contact, no significant difference in cell viability was observed between treatments, which may be due to the synergistic interaction resulting from rapid drug release and the internalization of cationic nanogels into cells (Fig 4D) [31]. It is also possible that the *in vitro* expression of CEA may be low compared to that of an *in vivo* system. Thus, additional studies are needed to fully explore the benefits of bioconjugation.

## 4. Conclusions

We investigated a system with potential applications for the development of site-specific anticancer therapies, which consisted of nanogels loaded with the anticancer drug 5-FU that were functionalized with shark VNARs. The nanogels were synthesized in a scalable, one-pot SFEP reaction and functionalized with an NHS-terminated polyethylene glycol derivative, which allowed for bioconjugation with shark VNARs. The system maintained its capacity to increase in size as pH decreased, with direct implications for the release kinetics of 5-FU. After bioconjugation, the ELISA results confirmed CEA recognition and the presence of VNAR. Subsequent *in vitro* evaluations indicated that the functionalized empty nanogels were not cytotoxic. Moreover, the cytotoxic effect of 5-FU was preserved after loading. A 15% reduction in cell viability was observed after two hours of contact with bioconjugated nanogels compared to what was observed with non-bioconjugated nanogels. Our results demonstrate the potential of this approach for the design of site-specific drug delivery systems. The conjugation strategy of the nanogels with VNAR was also used to effectively conjugate a Green fluorescent protein on the surface of nanogels with potential applications in theranostics. This is the first study to combine shark VNARs and nanogels to assess their potential for the development of anticancer therapies. In doing so, our results open up new avenues that may be explored to develop effective treatments for colon cancer and other diseases.

## Supporting information

S1 Raw images (PDF)

S1 File (PDF)

## References

[1] Fajardo-Ramirez O.R.; Ascacio-Martinez J.A.I.; Licea-Navarro A.F.; Villela-Martinez L.M.; Barrera-Saldana H.A. Technological Evolution in the Development of Therapeutic Antibodies. *Rev*. *Invest*. *Clin*. 2015, 67, 158–169. 26202739

[2] World Health Organization (2020). *Cancer Fact Sheet*. WHO Media Centre. Available online at: https://www.who.int/news-room/fact-sheets/detail/cancer.

[3] Global Burden of Disease 2019 Cancer Collaboration. Cancer Incidence, Mortality, Years of Life Lost, Years Lived With Disability, and Disability-Adjusted Life Years for 29 Cancer Groups From 2010 to 2019: A Systematic Analysis for the Global Burden of Disease Study 2019. *JAMA Oncol*. 2022, 8, 420–444. doi: 10.1001/jamaoncol.2021.6987 34967848 PMC8719276

[4] Moutabian H.; Majdaeen M.; Ghahramani-Asl R.; Yadollahi M.; Gharepapagh E.; Ataei G.; alahatpour Z.; Bagheri H., Farhood B. A systematic review of the therapeutic effects of resveratrol in combination with 5-fluorouracil during colorectal cancer treatment: with a special focus on the oxidant, apoptotic, and anti-inflammatory activities. *Cancer Cell*. *Int*. 2022, 22, 1–14. 35366874 10.1186/s12935-022-02561-7 PMC8976963

[5] Vinogradov S.V. Nanosized cationic hydrogels for drug delivery: preparation, properties and interactions with cells. *Adv. Drug Deliv*. *Rev*. 2002, 54, 135–147. doi: 10.1016/s0169-409x(01)00245-9 11755709

[6] Wesolowski J, Alzogaray V, Reyelt J, et al. Single domain antibodies: promising experimental and therapeutic tools in infection and immunity. *Med*. *Microbiol*. *Immunol*. 2009, 198, 157–74 doi: 10.1007/s00430-009-0116-7 19529959 PMC2714450

[7] Wondrak G.T. Redox-directed cancer therapeutics: molecular mechanisms and opportunities, Antioxid. *Redox Signal*. 2009, 11, 3013–3069. 10.1089/ars.2009.2541 PMC2824519 19496700

[8] Can M.; Guven O.; Sahiner N. Hacettepe J. Micro and nanogels for biomedical applications. *Biol*. *Chem*. 2020, 48, 407–424.

[9] Rejinold N. S.; Chennazhi K. P.; Nair S. V.; Tamura H.; Jayakumar R. Biodegradable and thermohermos-sensitive chitosan-g-poly(N-vinylcaprolactam) nanoparticles as a 5-fluorouracil carrier. *Carbohydr. Polym*. 2011, 83, 776–786.

[10] Cabanillas-Bernal O.; Dueñas S.; Ayala-Avila M.; Rucavado A, Escalante T.; Licea-Navarro, A.F. Synthetic libraries of shark VNAR domains with different cysteine numbers within the CDR3. *PloS One*, 2019, 14, e0213394. 31206542 10.1371/journal.pone.0213394 PMC6576789

[11] Adamo G.; Grimaldi N.; Sabatino M.A.; Walo M.; Dispenza C.; Ghersi G. E-beam crosslinked nanogels conjugated with monoclonal antibodies in targeting strategies. *Biol*. *Chem*. 2016, 398, 277–287. 10.1515/hsz-2016-0255 27508963

[12] Manzanares-Guevara L.A.; Licea-Claverie A.; Paraguay-Delgado F. Preparation of stimuli responsive nanogels based on poly(N,N-diethylaminoethyl methacrylate) by a simple “surfactant-free” methodology. *Soft Mater*. 2018, 16, 37–50.

[13] Ges Naranjo A.; Viltres Cobas H.; Kumar Gupta N.; Rodríguez López K.; Artimez Peña A.; Sacasas D.; Álvarez Brito R. 5-Fluorouracil uptake and release from pH-responsive nanogels: An experimental and computational study. *J*. *Mol*. *Liq*. 2022, 362, 119716.

[14] Peng H.; Huang H.; Shek P.; Charbonneau S.; Blostein M.D. PEGylation of Melittin: Structural Characterization and Hemostatic Effects. *Journal of Bioactive and Compatible Polymers* 2010, 25, 75–95.

[15] Manzanares-Guevara L.A.; Licea-Claverie A.; Oroz-Parra I.; Bernaldez-Sarabia J.; Diaz-Castillo F.; Licea-Navarro A.F. Smart nanoformulation based on stimuli-responsive nanogels and curcumin: Promising therapy against colon cancer. *ACS Omega* 2020, 5, 9171–9184. doi: 10.1021/acsomega.9b04390 32363269 PMC7191563

[16] Gruber A.; Navarro L.; Klinger D. Reactive Precursor Particles as Synthetic Platform for the Generation of Functional Nanoparticles, Nanogels, and Microgels *Adv*. *Mater*. *Interfaces* 2020, 7, 1901676.

[17] Borah P.K.; Rappolt M.; Duary R.K.; Sarkar A. Effects of folic acid esterification on the hierarchical structure of amylopectin corn starch, *Food Hydrocoll*. 2019, 86, 162–171.

[18] Chuacharoen T.; Sabliov S.M.; Zein nanoparticles as delivery systems for covalently linked and physically entrapped folic acid, *J*. *Nanopart*. *Res*. 2017, 19, 81–93.

[19] Hoffman R.M. Application of GFP imaging in cancer, *Lab*. *Invest*. 2015, 95, 432–452. doi: 10.1038/labinvest.2014.154 25686095 PMC4383682

[20] Jabr-Milane L. Multi-functional nanocarriers for targeted delivery of drugs and genes. *J*. *Control*. *Release*. 2008, 130, 121–128. doi: 10.1016/j.jconrel.2008.04.016 18538887

[21] Jabr-Milane L.S. Multi-functional nanocarriers to overcome tumor drug resistance. *Cancer Treat*. *Rev*. 2008, 34, 592–602. doi: 10.1016/j.ctrv.2008.04.003 18538481 PMC2585991

[22] Pignataro M.F.; Herrera M.G.; Dodero V.I. Evaluation of Peptide/Protein Self-Assembly and Aggregation by Spectroscopic Methods. *Molecules* 2020, 25, 4854. doi: 10.3390/molecules25204854 33096797 PMC7587993

[23] Hurwitz Herbert, Fehrenbacher L., Novotny W., Cartwright T., Hainsworth J., Heim W., Kabbinavar, F.Bevacizumab plus irinotecan, fluorouracil, and leucovorin for metastatic colorectal cancer. *New England Journal of Medicine*, 2004, 23, 2335–2342. 10.1056/NEJMoa032691 15175435

[24] Longley D., Harkin D. & Johnston P. 5-Fluorouracil: mechanisms of action and clinical strategies. *Nat*. *Rev*. *Cancer*, 2003, 3, 330–338. doi: 10.1038/nrc1074 12724731

[25] Xu Y.; Villalona-Calero M.A. Irinotecan: mechanisms of tumor resistance and novel strategies for modulating its activity, *Annals of Oncology*, 2002, 13, 1841–1851. doi: 10.1093/annonc/mdf337 12453851

[26] Clegg J.R; Sun J.A.; Gu J.; Venkataraman A.K.; Peppas N.A. Peptide conjugation enhances the cellular co-localization, but not endosomal escape, of modular poly(acrylamide-co-methacrylic acid) nanogels. *J*. *Control Release*. 2021, 329, 1162–1171. doi: 10.1016/j.jconrel.2020.10.045 33127451 PMC7904656

[27] Gallo E.; Diaferia C.; Smaldone G.; Morelli G.; Accardo A. Peptide-based hydrogels and nanogels for delivery of doxorubicin. *Int*. *J*. *Nanomed*. 2021, 16, 1617–1630. doi: 10.2147/IJN.S296272 33688182 PMC7935351

[28] Mohamed M.B.; Adbel-Ghani N.T.; El-Borady O.M.; and El-Sayed M.A. 5-Fluorouracil induces plasmonic coupling in gold nanospheres: new generation of chemotherapeutic agents. *J*. *Nanomed*. *Nanotechol*. 2012, 3, 146.

[29] Clogston J.D.; Patri A.K. “Zeta potential measurement.” Characterization of nanoparticles intended for drug delivery. Springer-Humana Press, 2011, 63–70. 10.1007/978-1-60327-198-1_6 21116954

[30] Manzanares-Guevara L. A.; Licea-Claverie A.; Oroz-Parra I.; Licea-Navarro A. F. On the cytotoxicity of a cationic tertiary amine PEGylated nanogel as nanocarrier for anticancer therapies. *MRS Commun*. 2018, 8, 1204−1210.

[31] González-Urías M.A.; Manzanares-Guevara L.A.; Zapata Gonzalez I.; Licea-Claverie A.; Bernaldez-Sarabia J.; Licea-Navarro A.F. Stimuli Responsive Nanogels with Intrinsic Fluorescence: Promising Nanovehicules for Controlled Drug Delivery and Cell Internalization Detection in Diverse Cancer Cell Lines. *Eur*. *Polym*. *J*., 2021, 144, 110200.

