# Supplementary figures and images for: Preparation of pH-sensitive nanogels bioconjugated with shark antibodies (VNAR) for targeted drug delivery with potential applications in colon cancer therapies
Source: PLoS One. 2024 Jan 19;19(1):e0294874. doi: 10.1371/journal.pone.0294874 (PMC10798631; doi:10.1371/journal.pone.0294874)

# S14 Fig. Raw Image

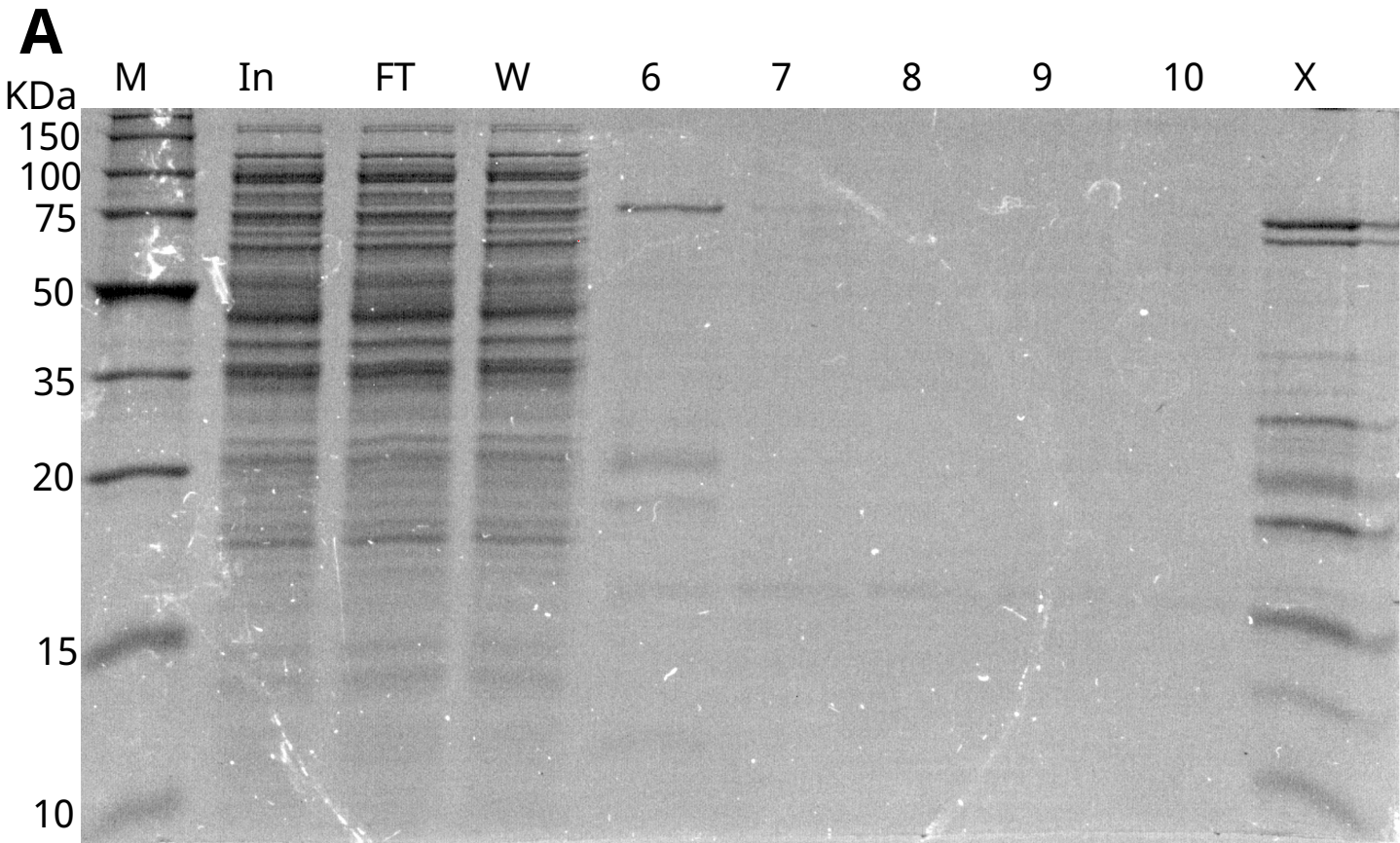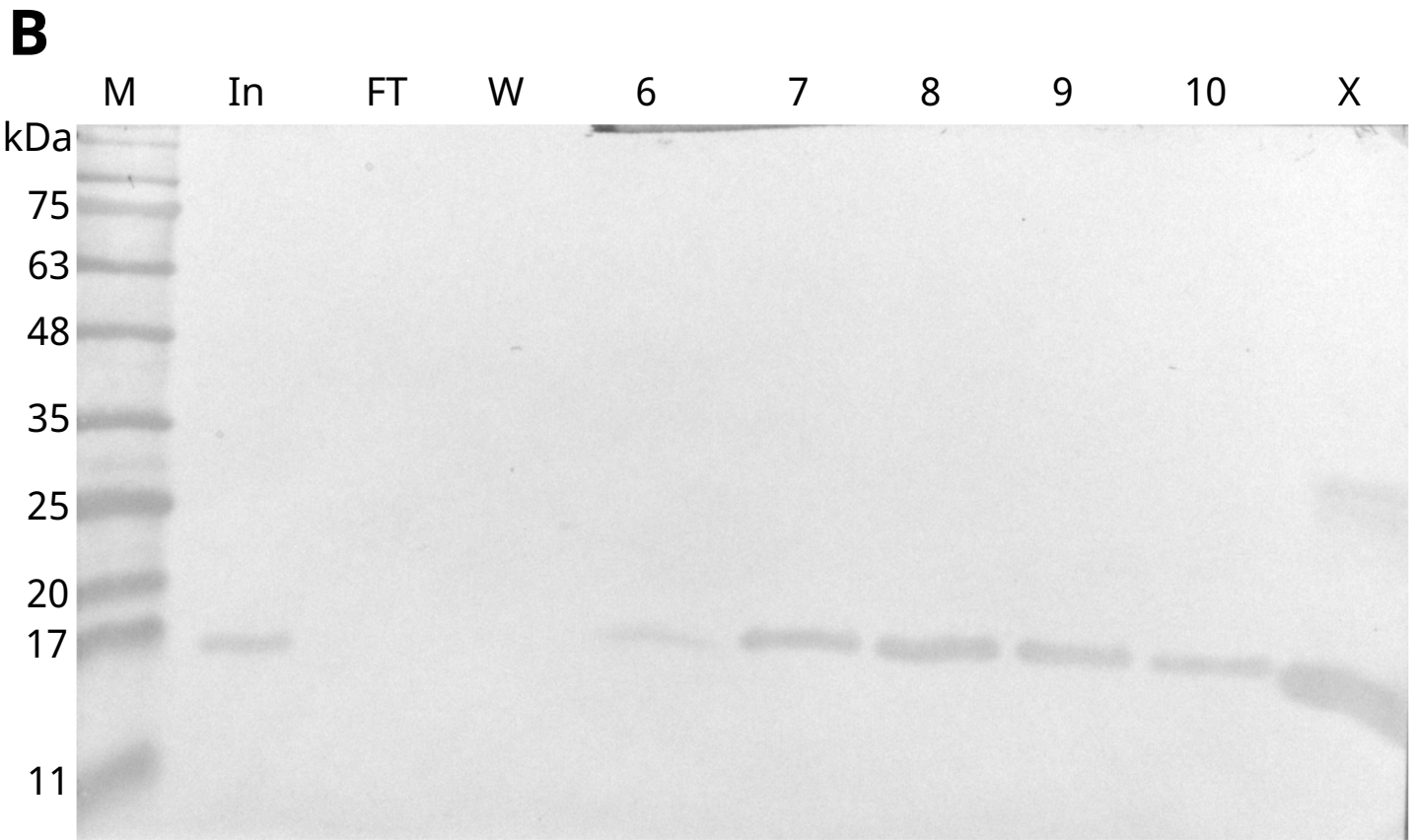

# S14 Fig. Raw Image

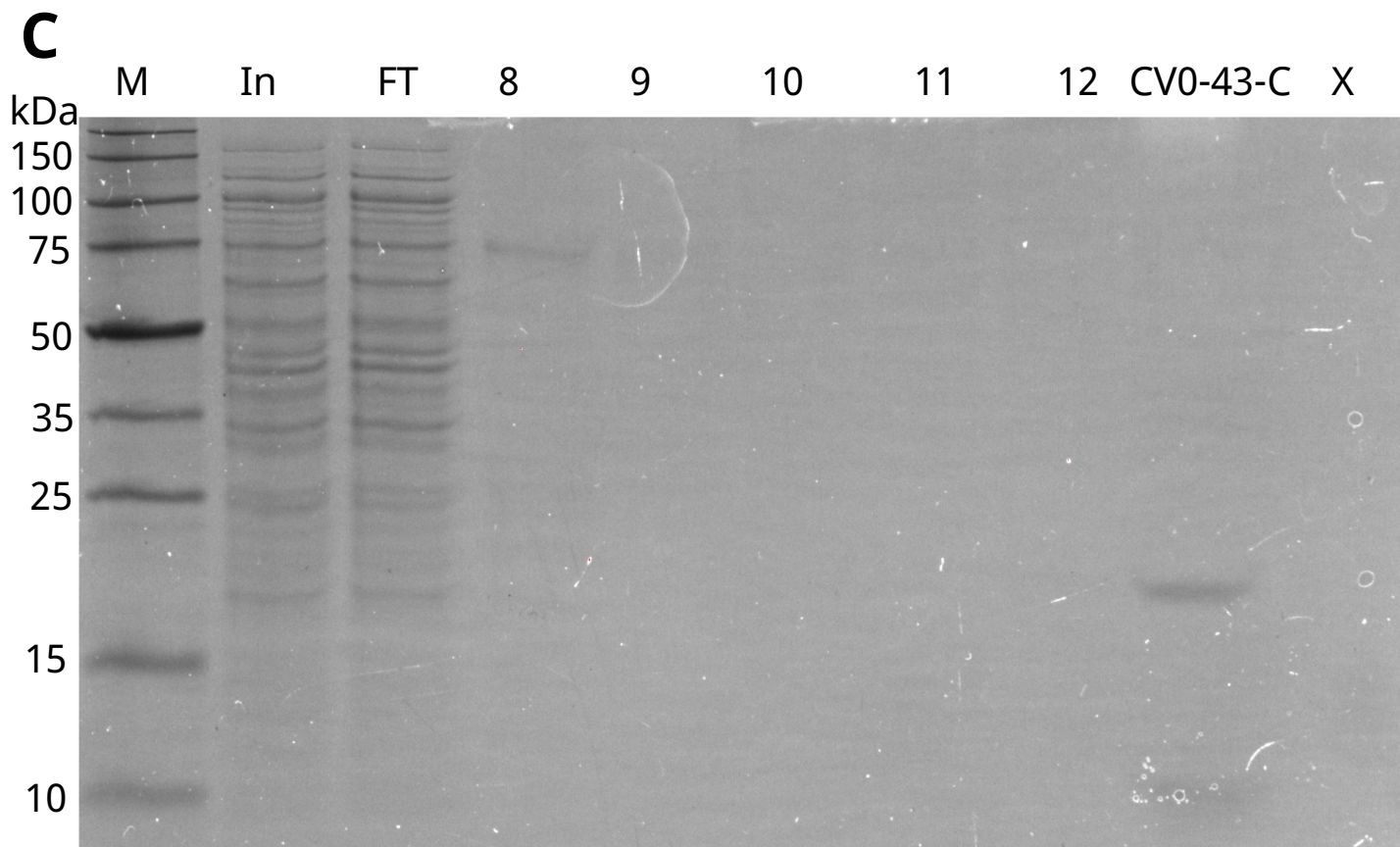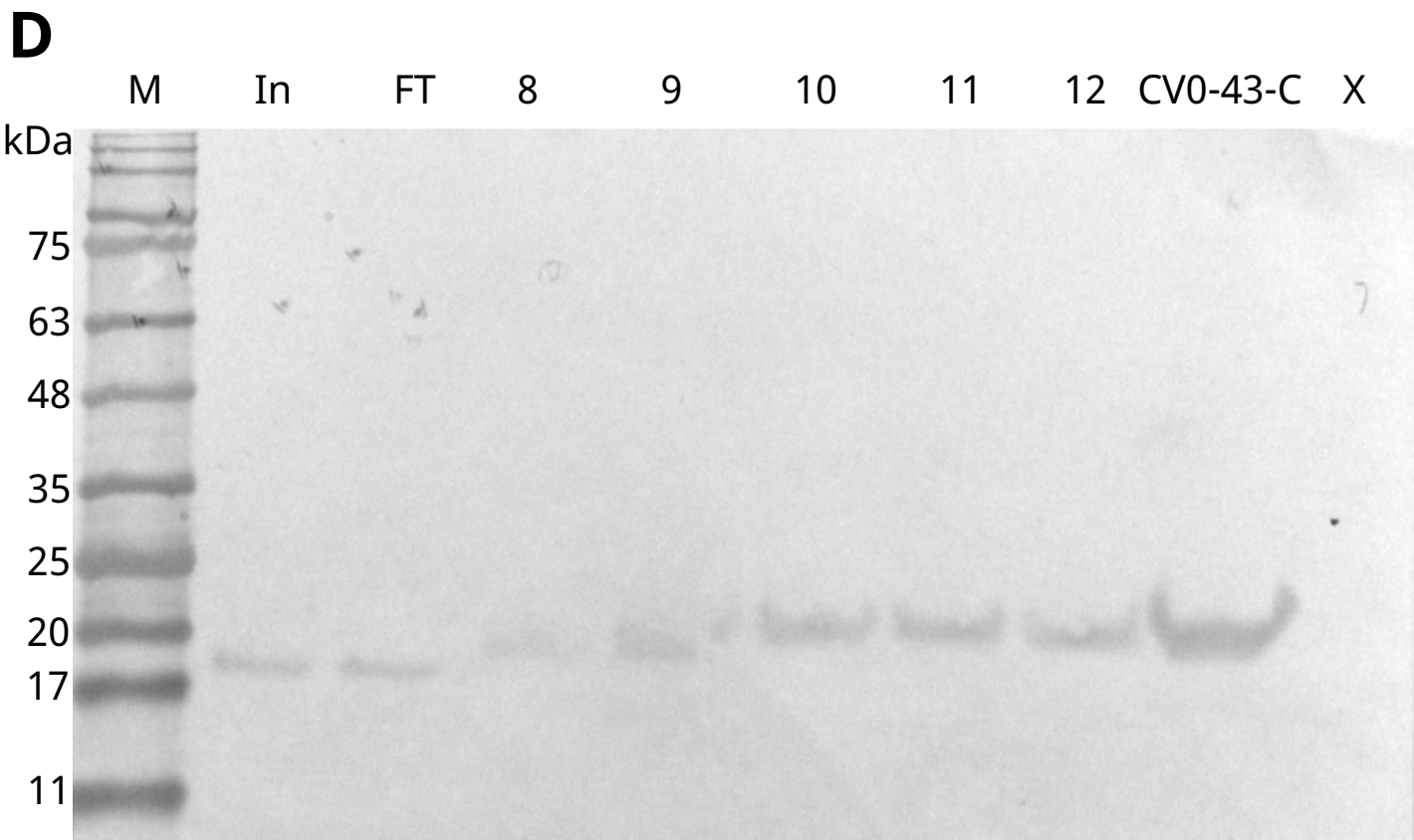

Supplement: S1 Raw images — (PDF) [file pone.0294874.s001.pdf]
