# Supplementary material for: Preparation of pH-sensitive nanogels bioconjugated with shark antibodies (VNAR) for targeted drug delivery with potential applications in colon cancer therapies
Source: PLoS One. 2024 Jan 19;19(1):e0294874. doi: 10.1371/journal.pone.0294874 (PMC10798631; doi:10.1371/journal.pone.0294874)
Supplement: S1 File — (PDF) [file pone.0294874.s002.pdf]

Preparation of pH-sensitive nanogels bioconjugated with shark antibodies (VNAR) for targeted drug delivery with potential applications in colon cancer therapies

Lizbeth A. Manzanares-Guevara, Jahaziel Gasperin-Bulbarela, Olivia Cabanillas-Bernal, Monserrat Renteria-Maciel, Angel Licea-Claverie, Eugenio R. Méndez, and Alexei F. Licea-Navarro

Supporting Information

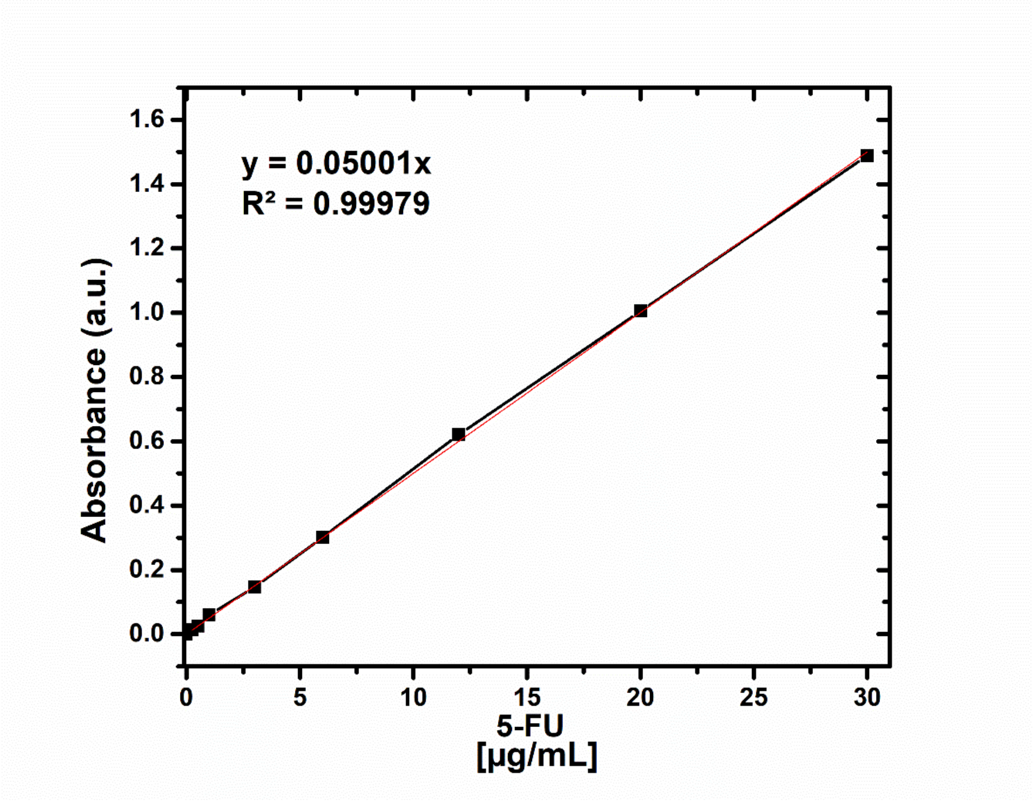

S1 Fig. Calibration curve of 5-FU at 266nm

Characterization of nanogels using nuclear magnetic resonance spectroscopy (^{1}H-NMR)

Given the fact that the amount of crosslinker used in the nanogels synthesis is less than 2 mol% of the DEAEM content and the DEAEM content is less than 70 % of the total mass content in the nanogels, then by ^{1}H-NMR it is hard to recognize the crosslinker. However the presence of PDEAEM, PEGMA and its content is clearly seen. The description of all signals and the composition determination is described for one example (N01), spectrum in S2 Fig:

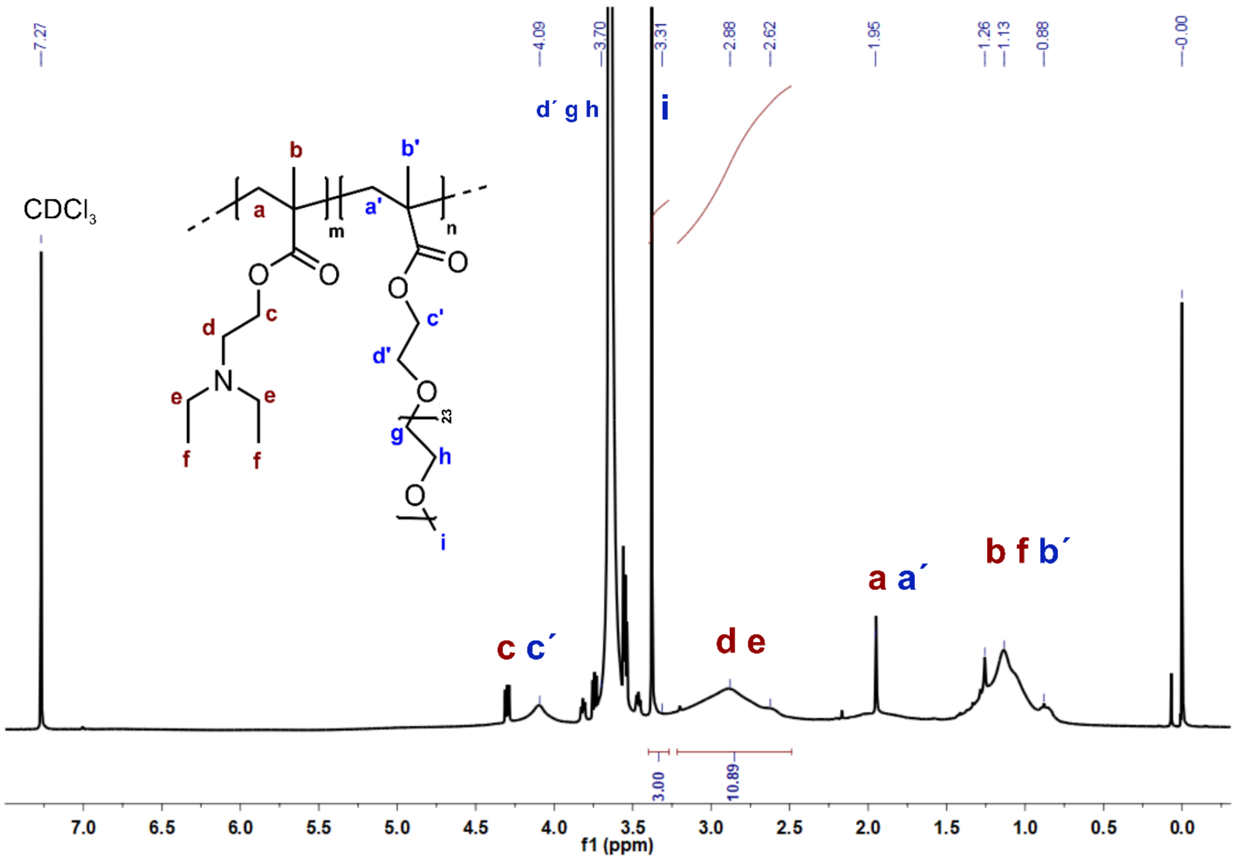

S2 Fig. ^{1}H-NMR spectrum of nanogels N01 in $CDCl_3$.

The signals between 0.9 and 1.4 ppm are assigned to the $-CH_3$ groups of the methacrylates (b=DEAEM and b'=PEGMA) and also to the hydrogens of the methyl-groups (f) of PDEAEM. The overlapping signal between 1.5 and 2.2 ppm corresponds to the $-CH_2-$ groups of the polymer backbone (a, a´); the signals between 2.5 and 3.2 ppm corresponds to the six hydrogens (d, e) of the methylene groups ($-N-CH_2-$) adjacent to the nitrogen of DEAEM; the signal at 3.31 ppm corresponds to the methyl hydrogens (i) of the methoxy end-group of PEGMA; the strong signal at 3.7 pm corresponds to the $-CH_2-O$ (d', g. h) of PEGMA; the signal at 4.1 ppm corresponds to two hydrogens (c) of the $–CH_2-O$ of the ester group of DEAEM and two hydrogens(c') of the $–CH_2-O$ of the ester group of PEGMA. The peak at 7.27 ppm corresponds to the deuterated solvent ($CDCl_3$). The composition was calculated by integration of signals at chemical shifts of 3.31 ppm (i) (3H) in the end group of the PEG side-chains and the integration of the signals between 2.5 and 3.2 ppm (d, e) (6H) of mehtylenes attached to the amine group of DEAEM as follows:

**PDEAEM:PEGMA (molar ratio) by ^{1}H-NMR**
PDEAEM: 6H =10.89 (integration); therefore, 1H = 1.81
PEGMA: 3H = 3 (integration); therefore, 1H=1.0
PDEAEM (1H)+PEGMA(1H)=2.81
**PDEAEM content (mol%)** =1.81/2.81=0.644 (mol), **64mol%**
**PEGMA content (mol%) =**1.0/2.81=0.355(mol), **36 mol%**

**PDEAEM:PEGMA (weight ratio) by ^{1}H-NMR**
PDEAEM (weight)=(0.644 mol)(185.27 g/mol)=119.31 g
PEGMA (weight)=(0.355 mol)(1000 g/mol)=355 g
PDEAEM (weight) +PEGMA (weight)=474.31 g
**PDEAEM(weight%)**=119.31 g/474.31 g =0.25(wt), **25 wt%**
**PEGMA(weight%)**=355 g/474.31 g=0.75(wt), **75 wt%**

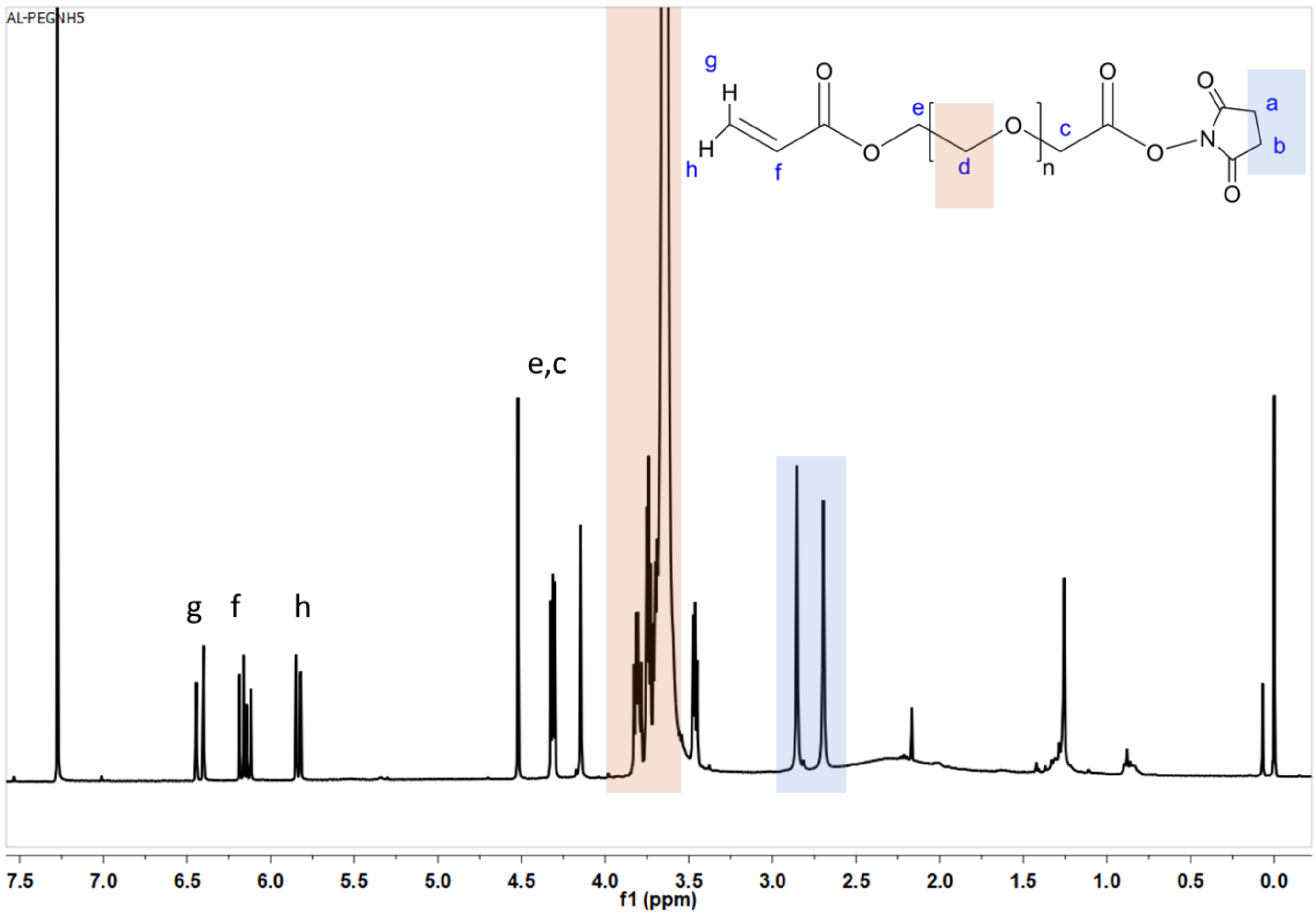

S3 Fig. ^{1}H-NMR spectrum of commercial PEGA3500-NHS in $CDCl_3$.

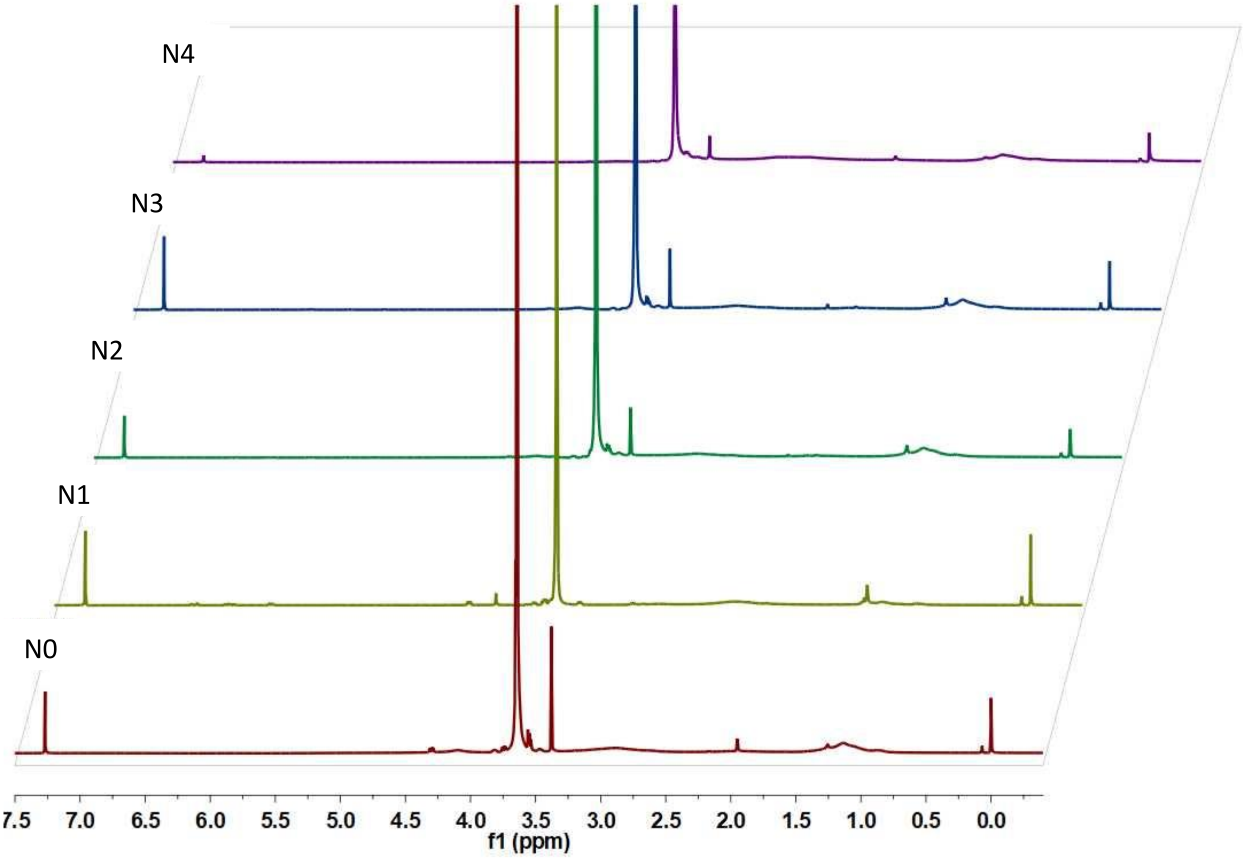

S4 Fig. ^{1}H-NMR spectrums of PDEAEM-based nanogels in $CDCl_3$.

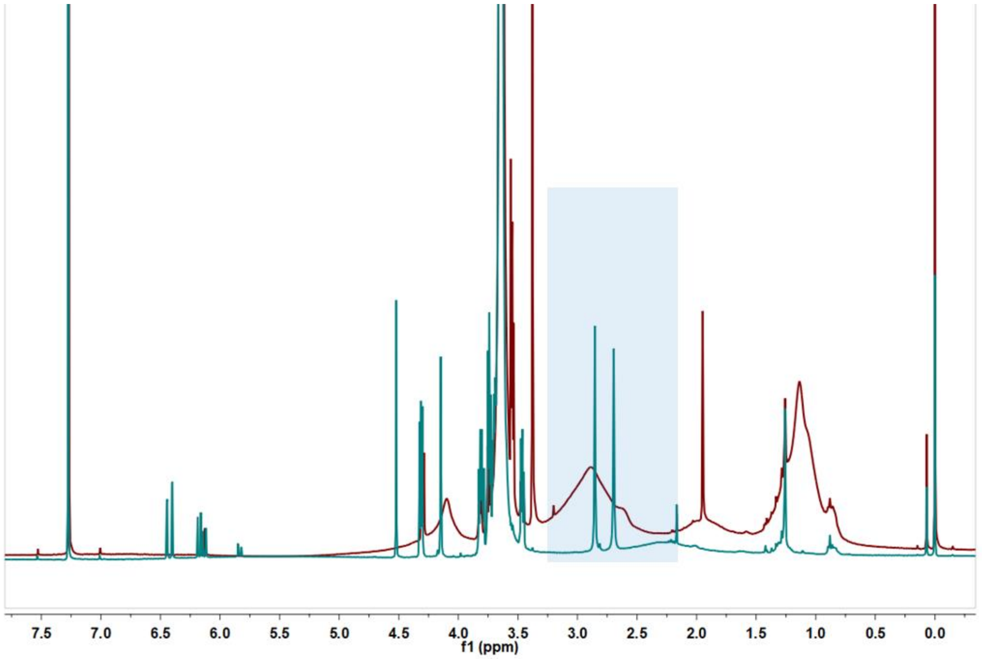

S5 Fig. ^{1}H-NMR spectrums of PEGA3500-NHS (blue) and PDEAEM-based nanogels (N0) in $CDCl_3$.

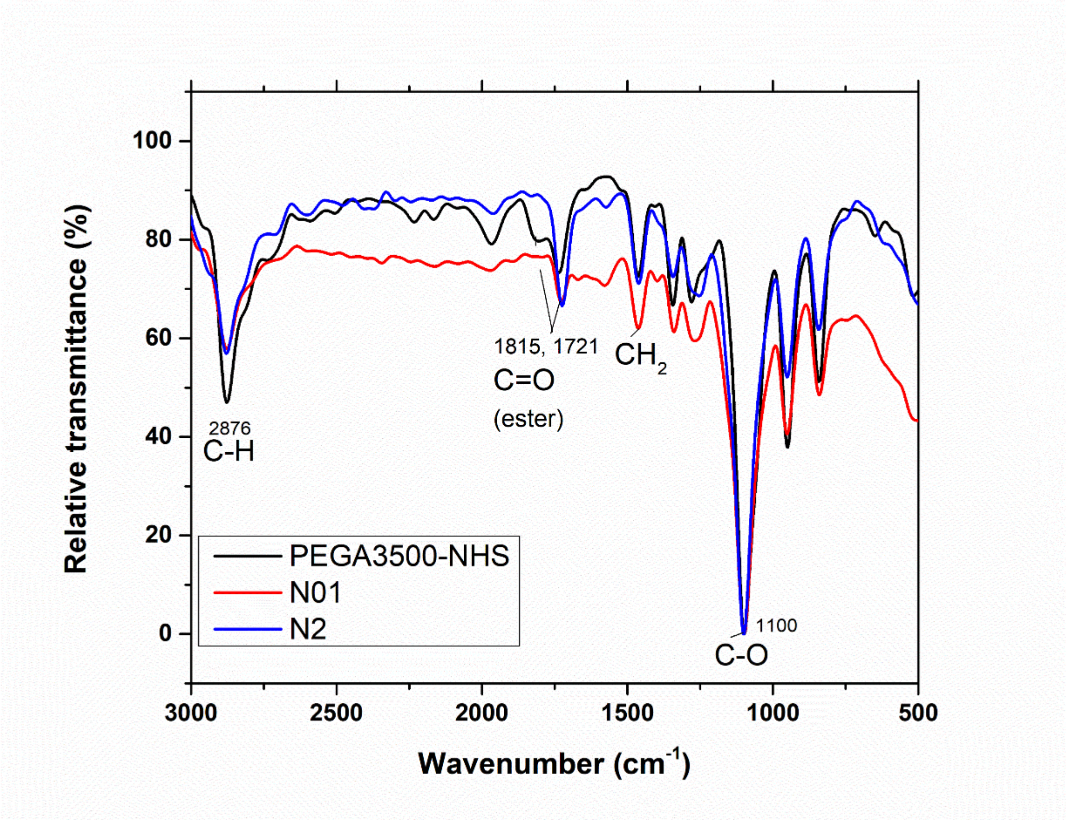

S6 Fig. FT-IR Spectra of PEGA3500-NHS, N01 and N2.

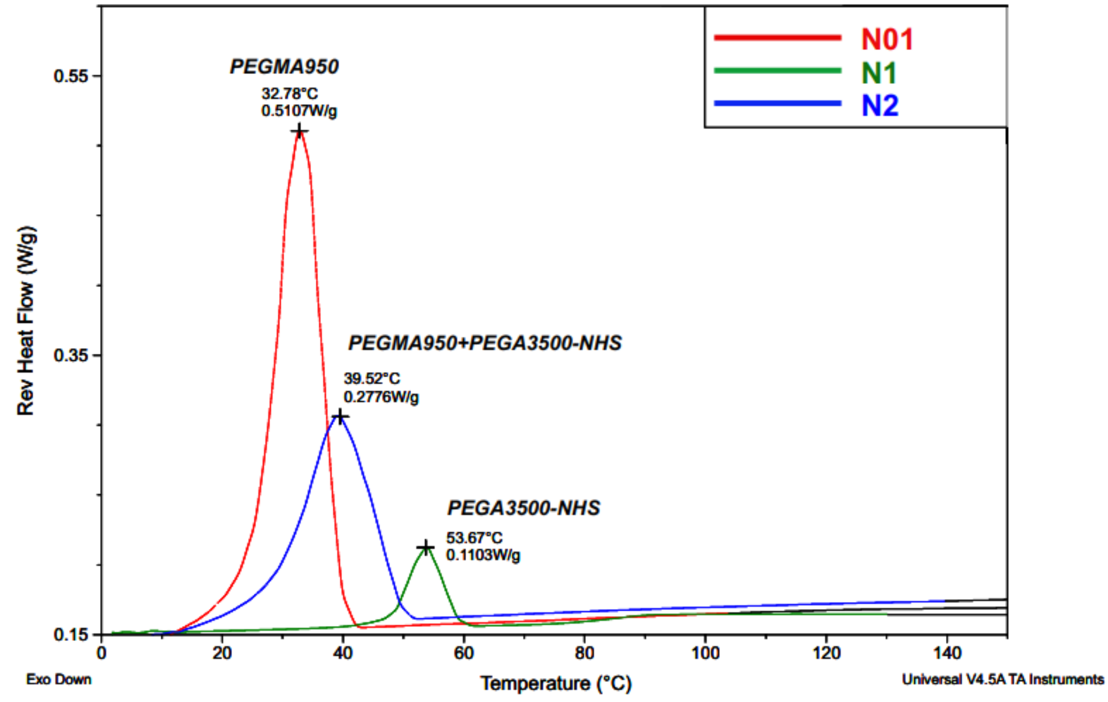

S7 Fig. Thermogram by DSC of PDEAEM-based nanogels, N01, N1 and N2.

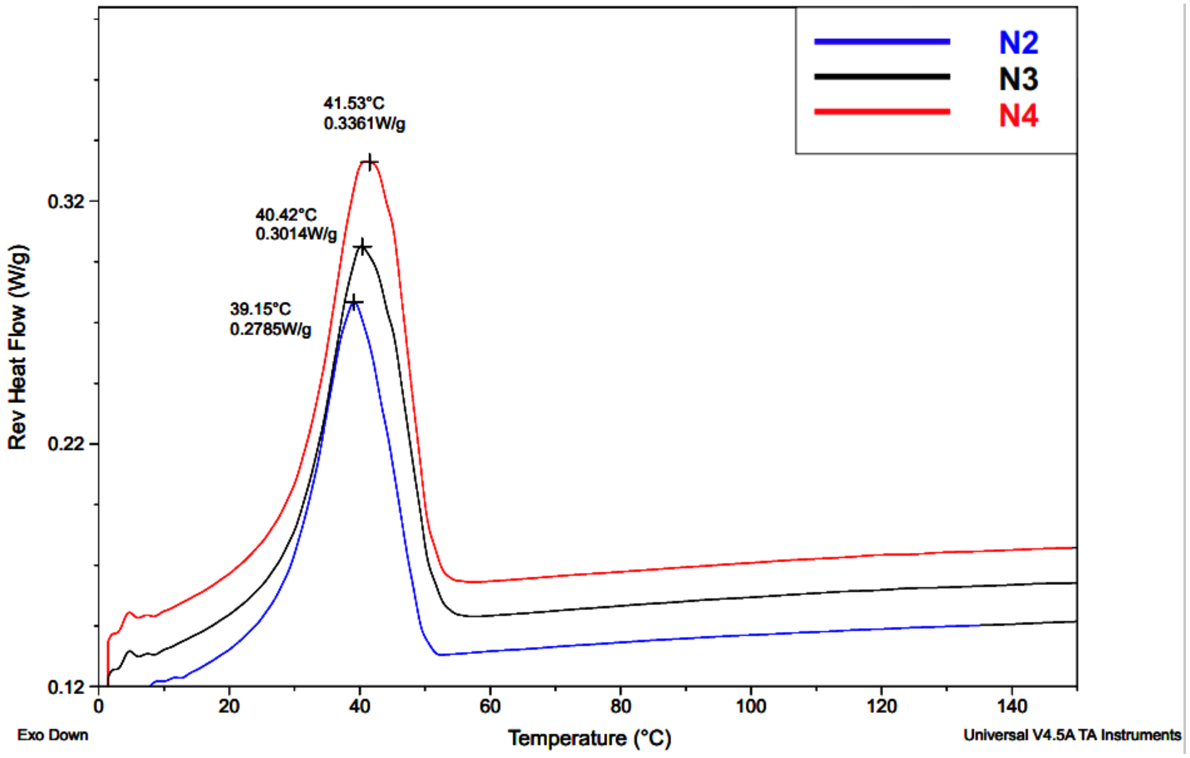

S8 Fig. Thermogram by DSC of PDEAEM-based nanogels, N2, N3 and N4.

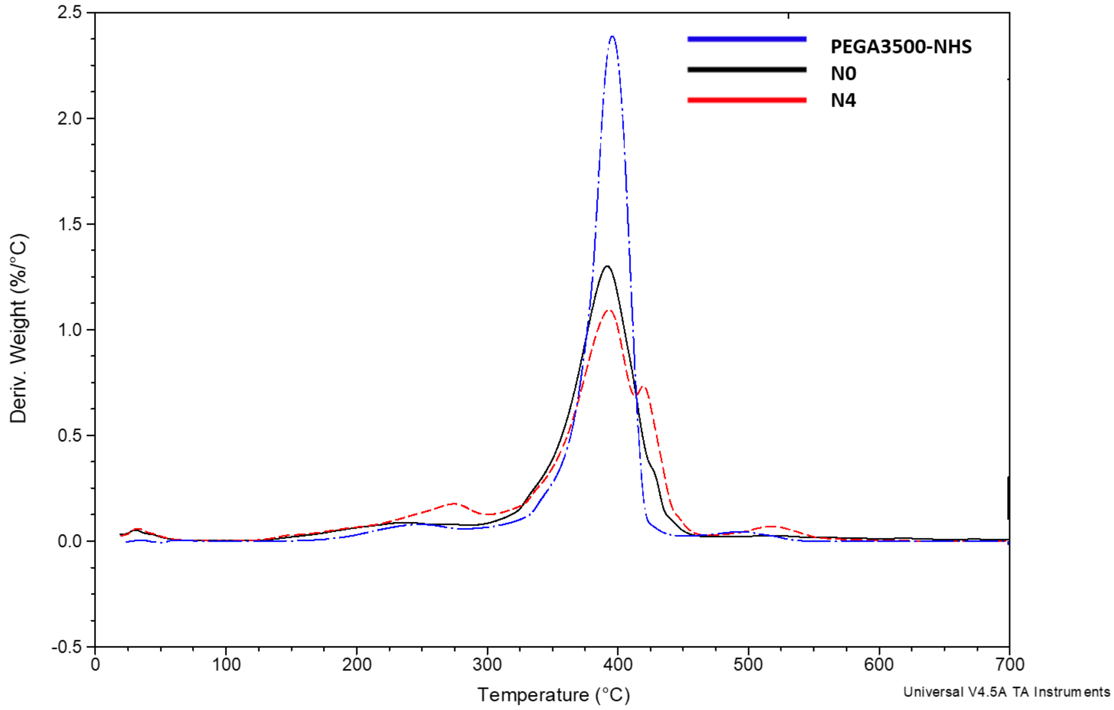

S9 Fig. Thermogram by TGA of PDEAEM-based nanogels, N2, N3 and N4.

S1 Table. Zeta potential of nanogels at different pH values

Nanogel	pH	ζ potential (mV)
**N0**	pH=5	+13.6
	pH=6.8	+11.2
	pH=7.4	+8.7
	pH=8	+3.5
**N3**	pH=5	+7.7
	pH=6.8	+5.1
	pH=7.4	+2.1
	pH=8	+2.7
**N6**	pH=5	+10.8
	pH=6.8	+5.5
	pH=7.4	+1.3
	pH=8	+0.8

a) Key	D_h by DLS (nm) 1X	PDI 1X	D_h by DLS (nm) 3X	PDI 3X
Non NHS functionalized				
N0	87	0.182	74	0.167
N01	102	0.158	90	0.097
[a]NHS Functionalized				
N2	118	0.105	102	0.031
N3	98	0.177	106	0.059
N4	110	0.103	103	0.057
N6	78	0.121	84	0.022

1X:0.5g/100 mL, 3X:1.5 g/300 mL

b)

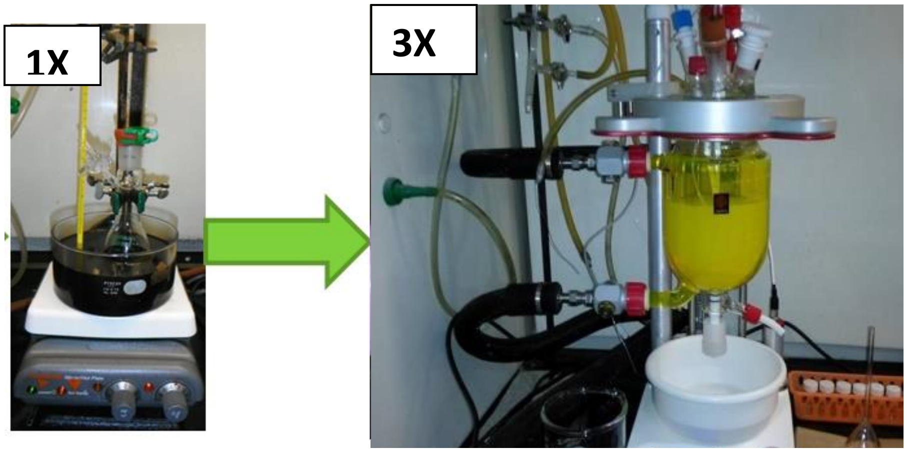

S10 Fig. Scalable process for synthesis of nanogels by SFEP, a) size values by DLS, b) photography of the synthesis 1X (50mL) and 3X (300 mL),

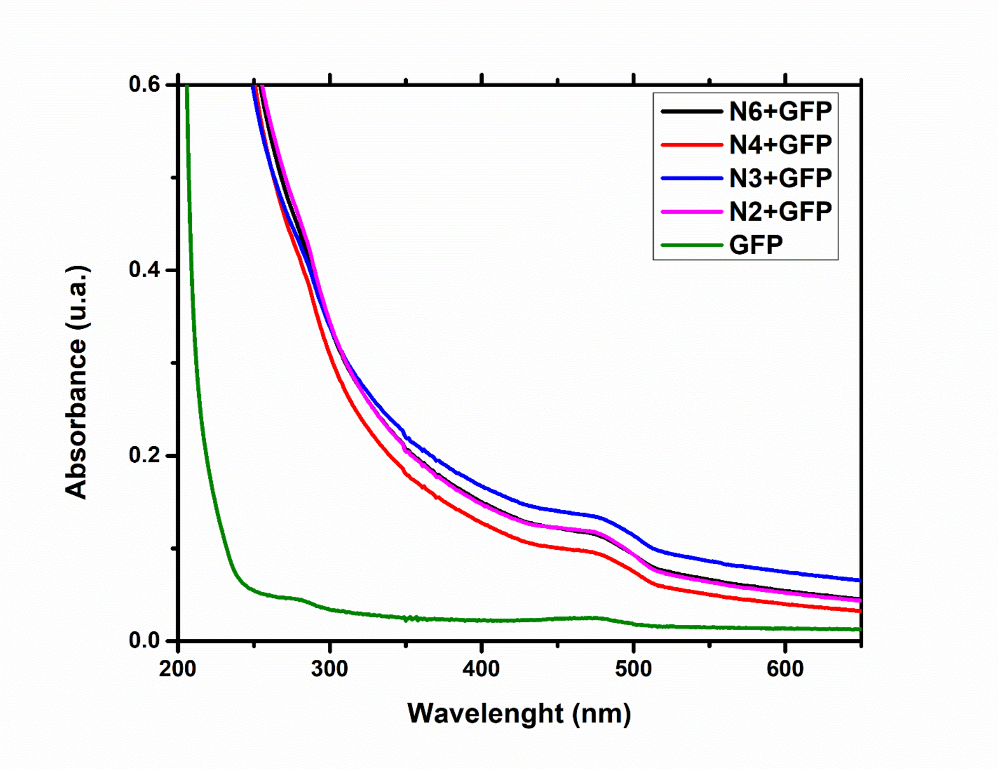

S11 Fig.UV-VIS Spectra for GFP and GFP-bioconjugated nanogels at PBS 7.4

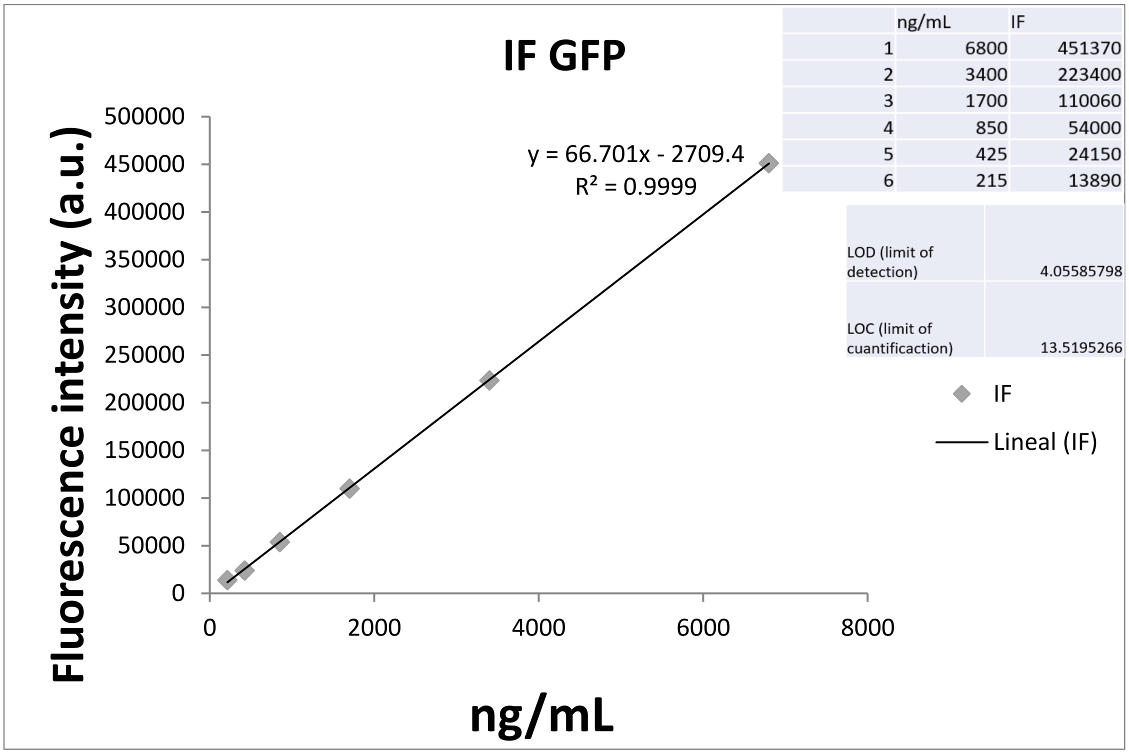

S12 Fig. Calibration curve of GFP at pH 7.4 by molecular fluorescence.

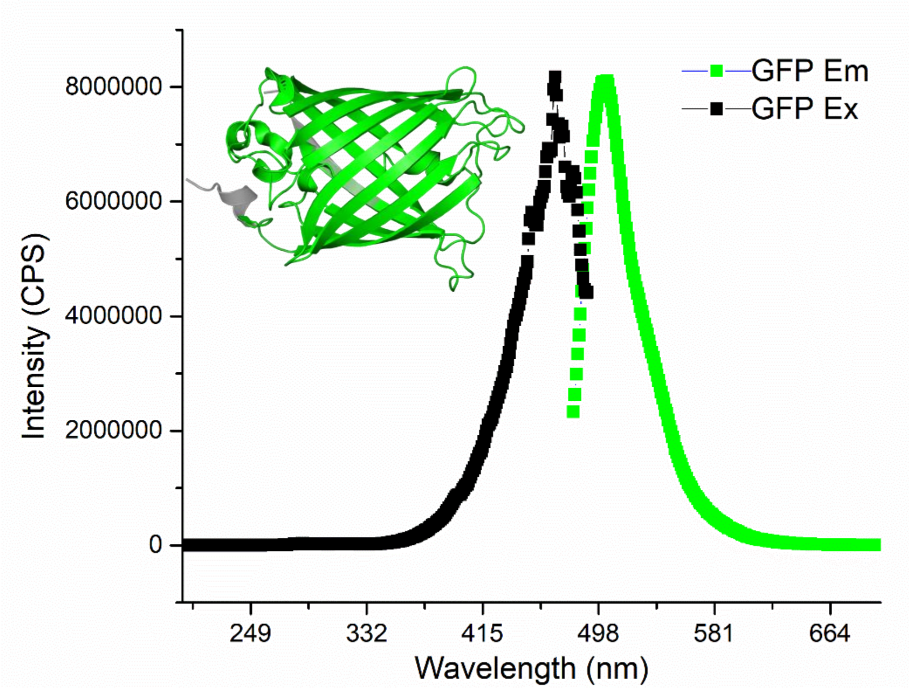

S13 Fig. Excitation and Emission of GFP at pH 7.4 by molecular fluorescence.

The VNAR CV043, which has recognition by the carcinoembryonic antigen (CEA), was expressed and purified with the following sequence:

ASLDQTPRTATRETGESLSINCVLTDTSHILFGTKWFWNNPGSTDWESITIGGRYVESVNNQAKSFSLQIKDLTVEDSGT YYCKA**DMVWSWWGGWRPVRRLGWKGWS**YYGAGTVLTVN**HHHHHH**GA**YPYDVPDYAS**LE

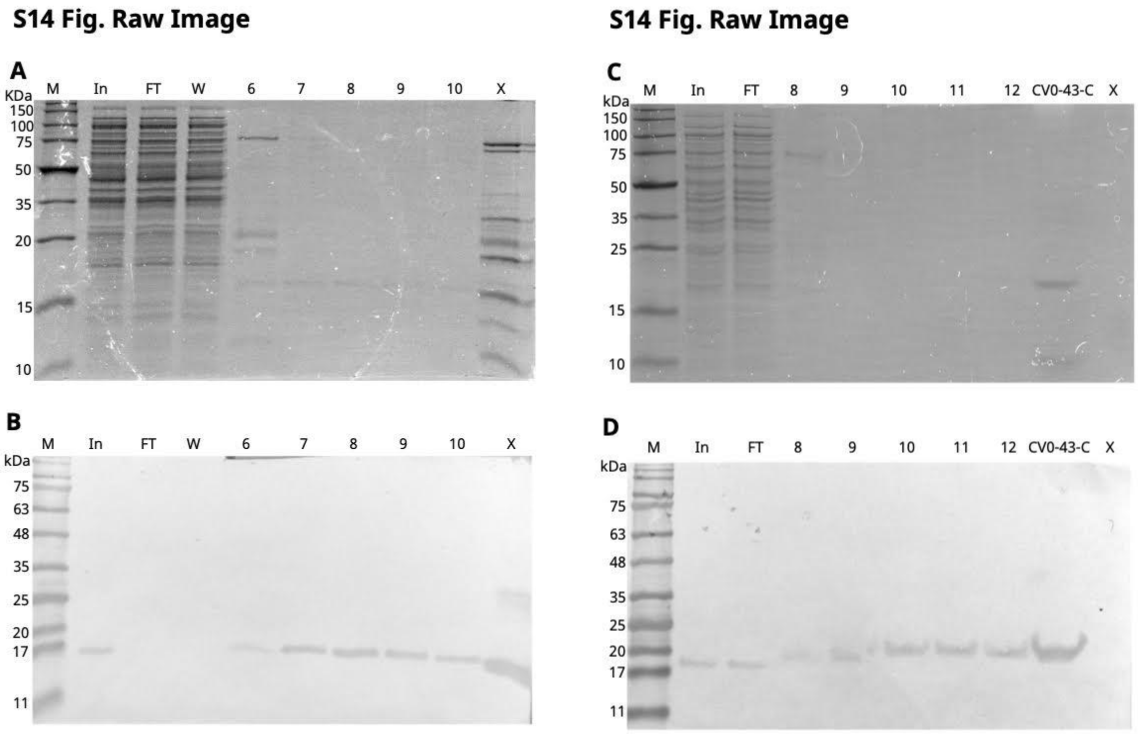

S14 Fig. Analysis of the purification of CV0-43. A & B correspond to Coomassie and Western Blot of the $MgSO_4$ supernatant, lane In is the initial sample before it passed through the column, FT is the sample after pass the column (flow through), W is the fraction of the wash step, lanes 6-10 are the elution fractions with buffer NPI500. C & D correspond to Coomassie and Western Blot of the Tris/saccharose supernatant, lane In is the initial sample, FT is flow through, lanes 8-12 are the elution fractions with buffer NPI500, and CV0-43C is the concentration of the fractions 8-10 from de MgSO4 supernatant purified. The lane M is the molecular weight standard, Protein Broad Range (Promega) for Coomassie and Opti-Protein XL Marker for Western Blot. The weight of CV0-43 is 15.7 kDa.

The purity of the protein can be seen in the figures S12A (lanes 8-10) and S12C (lanes 9-12), and the figures S12B (8-10) and S12D (10-12) shows the fractions with the higher presence of CV0-43. The concentration of the protein purified, in the lane CV0-43-C in figure S12C and S12D, was 350 µg/ml.

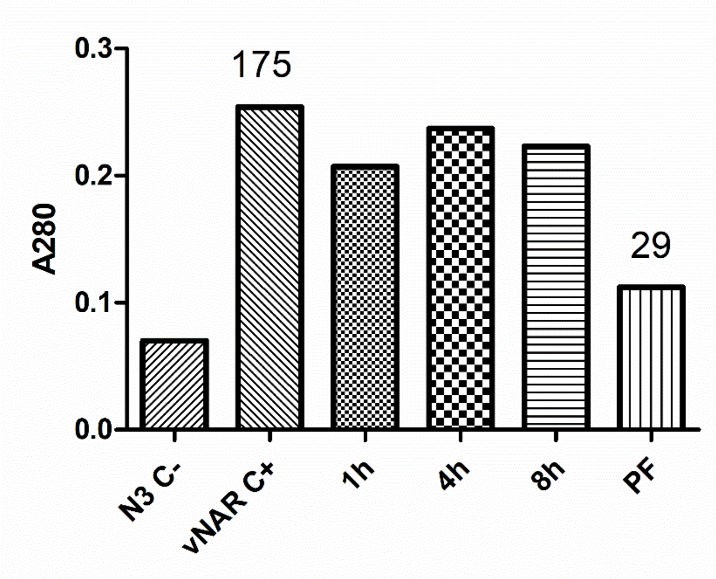

S15 Fig. UV-VIS at 280 nm, N3 (nanogel without vNAR), vNAR (175 µg/ml), PF (N3+vNAR after dialysis 29 µg/ml, subtracting the absorbance of N3).

5FU showed cytotoxic effect on HCT-116 (Human colorectal cancer, IC50: 125 µg/mL, 24h).

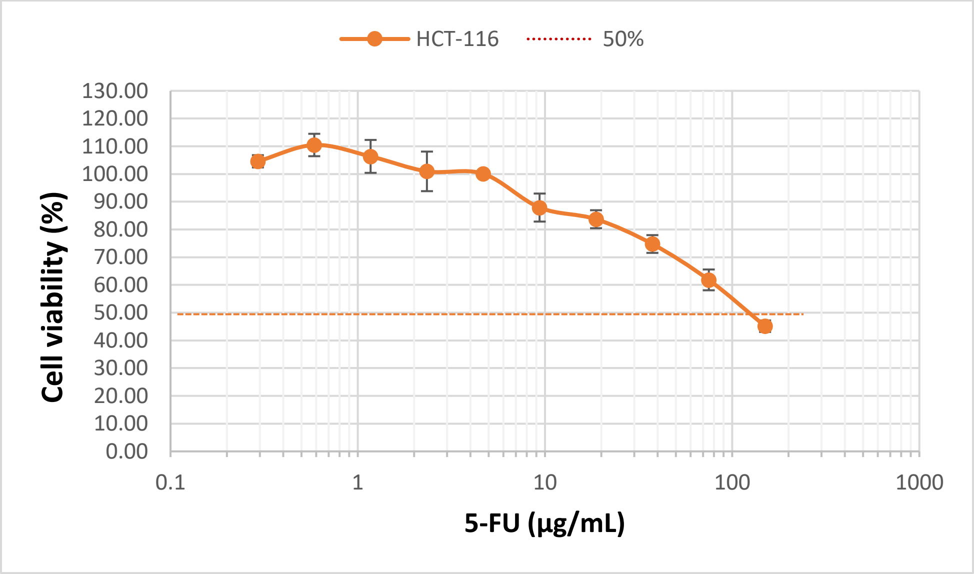

S16 Fig. Cell viability by MTS of 5FU in HCT-116 (orange line).
